# Differential loss of effector genes in three recently expanded pandemic clonal lineages of the rice blast fungus

**DOI:** 10.1186/s12915-020-00818-z

**Published:** 2020-07-16

**Authors:** Sergio M. Latorre, C. Sarai Reyes-Avila, Angus Malmgren, Joe Win, Sophien Kamoun, Hernán A. Burbano

**Affiliations:** 1 Research Group for Ancient Genomics and Evolution, Max Planck Institute for Developmental Biology, Tuebingen, Germany; 2 The Sainsbury Laboratory, University of East Anglia, Norwich Research Park, Norwich, UK; 3 Natural History Museum of Denmark, University of Copenhagen, Copenhagen, Denmark; 4 Centre for Life’s Origin and Evolution, Department of Genetics, Evolution and Environment, University College London, London, UK

**Keywords:** Fungi, Pathogens, Plants, Rice, Cereals, Genomes, Population history, Effectors, Infectious diseases, Pandemics

## Abstract

**Background:**

Understanding the mechanisms and timescales of plant pathogen outbreaks requires a detailed genome-scale analysis of their population history. The fungus *Magnaporthe* (Syn. *Pyricularia*) *oryzae*—the causal agent of blast disease of cereals— is among the most destructive plant pathogens to world agriculture and a major threat to the production of rice, wheat, and other cereals. Although *M. oryzae* is a multihost pathogen that infects more than 50 species of cereals and grasses, all rice-infecting isolates belong to a single genetically defined lineage. Here, we combined the two largest genomic datasets to reconstruct the genetic history of the rice-infecting lineage of *M. oryzae* based on 131 isolates from 21 countries.

**Results:**

The global population of the rice blast fungus consists mainly of three well-defined genetic groups and a diverse set of individuals. Multiple population genetic tests revealed that the rice-infecting lineage of the blast fungus probably originated from a recombining diverse group in Southeast Asia followed by three independent clonal expansions that took place over the last ~ 200 years. Patterns of allele sharing identified a subpopulation from the recombining diverse group that introgressed with one of the clonal lineages before its global expansion. Remarkably, the four genetic lineages of the rice blast fungus vary in the number and patterns of presence and absence of candidate effector genes. These genes encode secreted proteins that modulate plant defense and allow pathogen colonization. In particular, clonal lineages carry a reduced repertoire of effector genes compared with the diverse group, and specific combinations of presence and absence of effector genes define each of the pandemic clonal lineages.

**Conclusions:**

Our analyses reconstruct the genetic history of the rice-infecting lineage of *M. oryzae* revealing three clonal lineages associated with rice blast pandemics. Each of these lineages displays a specific pattern of presence and absence of effector genes that may have shaped their adaptation to the rice host and their evolutionary history.

## Background

Plant diseases are a persistent threat to food production due to a notable increase in the emergence and spread of new pathogens [1, 2]. Understanding the mechanisms and timescales associated with new epidemics is essential for both basic studies and the implementation of effective response measures [3]. A fundamental component of this knowledge is a detailed genome-scale understanding of the population structure and dynamics of global plant pathogen populations [4–6]. Population genetic information drives the selection of isolates for activities as diverse as basic mechanistic research and plant germplasm screening for disease resistance. It also helps to pinpoint the origin of pandemic strains and the evolutionary potential of different pathogen populations [7–12]. A thorough understanding of the global population structure is essential for any surveillance program that aims at rapidly detecting pathogen incursions into new geographical areas. In addition, the recent knowledge gained in the biology of pathogen effectors—secreted molecules that modulate host responses—brings yet another dimension to the population genetics framework, as it enables the reconstruction of the evolutionary history of virulence traits and helps guide the deployment of disease-resistant cultivars [7, 13–16].

Fungal plant pathogens account for ~ 10–80% of crop losses in agriculture and are viewed as a major threat to global food security [1, 2, 17, 18]. Cereal crops like rice, oat, millet, barley, and wheat have provided the foundation of modern agriculture and the success of humankind. Today’s agriculture is facing the challenge of ensuring global food security for an ever-expanding world population, which is estimated to exceed 9 billion within the next 30 years [19]. The ascomycete fungus *Magnaporthe* (Syn. *Pyricularia*) *oryzae*, the causal agent of blast disease of cereals, is often ranked as the most destructive fungal pathogen, causing losses in rice production that, if mitigated, could feed several hundred million people [1, 20]. Despite its Linnean name, *M. oryzae* is a multihost pathogen that can also cause the blast disease on other cereal crops, notably on wheat where it has recently spread from South America to Bangladesh resulting in destructive outbreaks [8, 21, 22]. *M. oryzae* reproduces mainly asexually and field isolates of *M. oryzae* are haploid. Asexual reproduction is the predominant mode of reproduction in almost all rice-growing regions; however, population genetics evidence has identified sexually reproducing populations in Southeast Asia, indicating that *M. oryzae* likely lost sexual reproduction outside of its center of origin [23].

Comparative genomics analyses provided insights into the population structure and host-specialization of *M. oryzae* [24–26]. This pathogen consists of a complex assemblage of genetically distinct lineages that tend to be associated with particular host genera [26]. Remarkably, all rice-infecting isolates belong to a single genetic lineage that is thought to have originated from isolates infecting foxtail millet (*Setaria italica* and *Setaria viridis*). *M. oryzae* host-specific lineages exhibit limited gene flow but recurrent gene gain/loss particularly in regions of the genome linked to transposable elements [24, 25]. As in many other plant pathogens, effector genes exhibit a high degree of presence and absence polymorphisms and signatures of adaptive evolution (e.g., higher rate of non-synonymous over synonymous mutations) [25]. Loss of so-called AVR effector genes—activators of host immunoresponses—can dramatically impact the fitness of the blast fungus by enabling virulence on resistant host genotypes [22, 27, 28].

Although the genome sequence of the *M. oryzae* strain 70-15 was at the time of its publication the first fungal plant pathogen genome to be described [29], it took about a decade before comparative genomics analyses of this pathogen started to be reported [24, 25, 30]. Until recently, understanding of the population genomics structure of the rice blast fungus has remained limited. In 2018, two studies reported whole genome sequences from non-overlapping sets of globally distributed rice-infecting *M. oryzae* isolates [31, 32]. Both studies suggested the presence of a diverse Southeast Asian population and two major clonal groups. However, due to sampling or analytical limitations the two studies reached different conclusions about the composition of worldwide populations, i.e., the number of genetic groups and the processes that gave rise to them.

Here, we performed a combined analysis that builds on the studies of Gladieux et al. [31] and Zhong et al. [32] to reconcile the two datasets and increase the number of examined *M. oryzae* individuals to 131 isolates from 21 countries. This has enabled us to assess the global genetic structure of rice-infecting *M. oryzae* more comprehensively than the prior separate analyses of the two datasets. We discovered that the global population of the rice blast fungus consists mainly of three well-defined genetic groups and a diverse set of individuals. Multiple population genetic tests revealed that the rice blast fungus probably originated from a recombining population in Southeast Asia followed by three independent clonal expansions that took place over the last ~ 100–200 years. Patterns of allele sharing identified a subpopulation from the recombining group that introgressed with one of the clonal lineages before its global expansion. Remarkably, the genetic lineages of the rice blast fungus vary in the number and patterns of presence and absence of secreted protein predicted as effectors. In particular, the clonal lineages are defined by specific sets of effectors that may have shaped their adaptation to the rice host and their evolutionary history.

## Results and discussion

### The global population structure of rice-infecting *Magnaporthe oryzae* consists of three well-defined genetic groups and a diverse set of individuals

To assess the global population structure of rice-infecting *M. oryzae*, we used a total of 131 genome sequences from Gladieux et al. (*N* = 43) [31] and Zhong et al. (*N* = 88) [32]. The combined use of samples from these two studies increases not only the number of *M. oryzae* samples but also their geographical spread (Additional file 1: Fig. S2B-C and Additional file 2: Table S1). We identified a total of 39,862 single-nucleotide polymorphism (SNPs) (see the “Methods” section). For subsequent analyses, we only used SNPs ascertained in all samples (“full information”) (*N* = 11,478 SNPs).

**Fig. 1.**
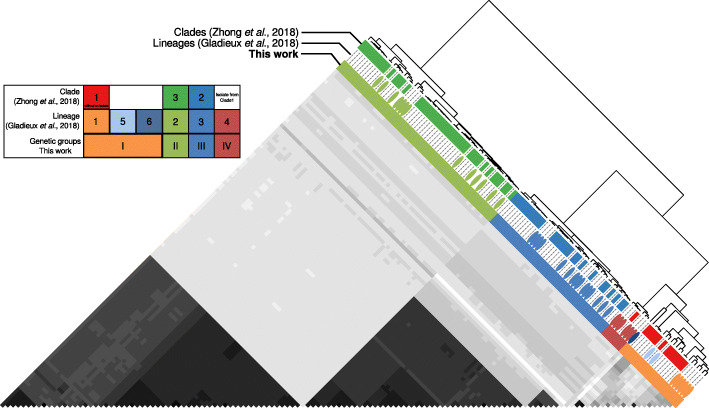
Genetic clustering of *Magnaporthe oryzae* reveals three defined groups and a diverse set of individuals. The pairwise relatedness between *M. oryzae* samples (X and Y) was estimated using f3-outgroup statistics of the form *f3*(X, Y; outgroup), which measures the amount of shared genetic history (genetic drift) between X and Y after the divergence from an outgroup (*M. oryzae* strain from *Setaria*). The hierarchical clustering is based on *f3-scores* resulting from f3-outgroup statistic calculations. Darker colors indicate more shared drift

We first sought to investigate the number of distinct genetic groups in our global sample of *M. oryzae* given previous discrepancies in the number of clades or lineages identified in the two studies. We identified three well-defined groups and a diverse set of individuals based on two lines of evidence. First, we used f3-outgroup statistics [33] to evaluate the pairwise relatedness between *M. oryzae* samples relative to an outgroup. The f3-outgroup statistics measure the amount of shared evolutionary history between samples, which can be interpreted as shared genetic drift (always relative to an outgroup). We summarized the results of all tests by performing hierarchical clustering based on pairwise shared genetic drift comparisons, i.e., *z*-scores derived from f3-outgroup statistic tests (Fig. 1). Additionally, we calculated pairwise Hamming genetic distances between all samples and summarized the information using principal component analysis (PCA). The samples clustered again in three distinct groups and one diverse set of individuals using PC1, 2, and 3, which together explained more than 90% of the variance (Additional file 1: Fig. S1A). We assessed the robustness of these clusters using Silhouette scores, which indicate how similar an individual is to its own cluster compared to other clusters [34]. We found that the best mean Silhouette scores were obtained when the dataset was divided into four clusters (Additional file 1: Fig. S1B).

**Fig. 2.**
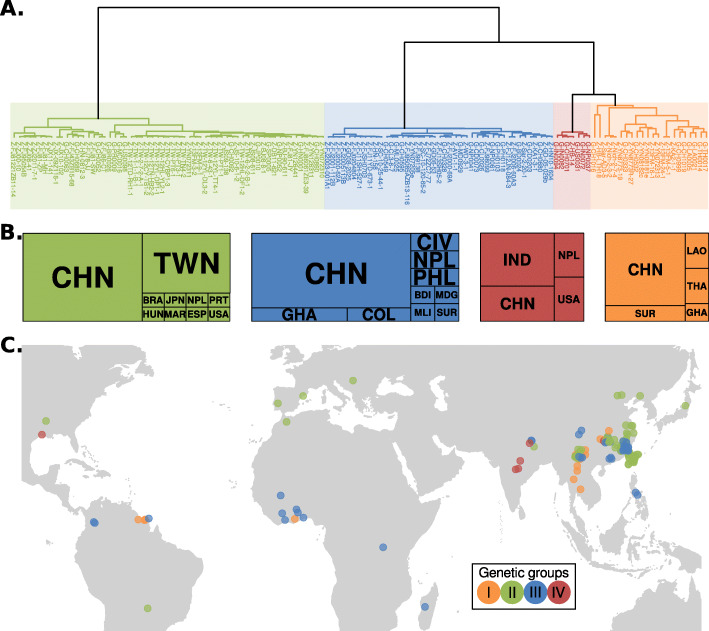
Geographic location of *Magnaporthe oryzae* isolates shows global distribution of defined genetic groups (II–III) and a preferential Southeast Asian location for the diverse group (I). **a** Dendrogram showing the hierarchical clustering based on pairwise f3 values (same as Fig. 1). Prefixes of the isolate names correspond to the database source: G = Gladieux et al., 2018 [31]; Z = Zhong et al., 2018 [32]. **b** Country of origin for *M. oryzae* isolates. The overall size of the boxes represents the total number of samples within each genetic group. The size of each internal box is proportional to the number of isolates per country. Countries are represented as three-letter codes (ISO 3166-1 alpha-3): BDI = Burundi, BRA = Brazil, CHN = China, CIV = Côte d’Ivoire, COL = Colombia, ESP = Spain, GHA = Ghana, HUN = Hungary, IND = India, JPN = Japan, LAO = Lao People’s Democratic Republic, MAR = Morocco, MDG = Madagascar, MLI = Mali, NPL = Nepal, PHL = Philippines, PRT = Portugal, SUR = Suriname, THA = Thailand, TWN = Taiwan, Province of China, USA = United States of America. **c** Geographical origin of samples used in this study. A random jitter was used on the coordinates of geographical-close samples for better visualization

Since our two approaches consistently revealed the presence of four groups, we named them groups I, II, III, and IV. Whereas groups II and III are geographically widespread, group I is mainly located in Southeast Asia and group IV in the Indian subcontinent (Fig. 2). The correspondence between our classification and previously described nomenclatures can be found in Additional file 3: Table S2. Our grouping very likely recapitulates the four lineages proposed by Saleh et al. based on microsatellite data [35]. Although it is not possible to directly link the microsatellite data with our analysis, we linked the correspondence between groups indirectly through the analysis presented in Gladieux et al., the same group that previously performed the microsatellite analysis. Zhong et al. [32] divided their dataset in three groups (I–III) but did not identify group IV, since their dataset only included one individual from this group. In addition to groups I–IV, Gladieux et al. [31] identified two additional lineages based on a set of phylogenetic analyses. The combined analysis presented here showed that these additional lineages from Gladieux et al. are within the genetic diversity of group I, thus splitting of group I is not warranted.

### Global population of rice-infecting *Magnaporthe oryzae* probably arose from a recombining Southeast Asian population followed by clonal expansions

**Fig. 3.**
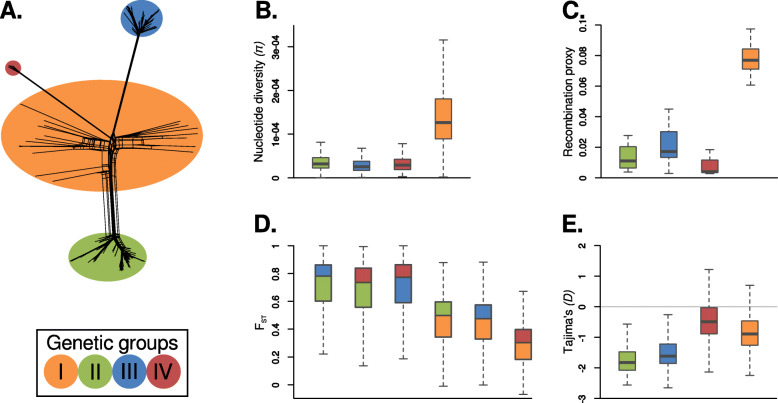
*Magnaporthe oryzae* population structure is driven by recombination and global clonal expansions. **a** Phylogenetic network showing the three well-defined groups (green, blue, and red) and the diverse set of individuals (orange) from Fig. 1. **b** Within-population comparisons of nucleotide diversity measured as π. **c** Recombination proxy calculated by dividing the number of violations of the four-gamete test by the total number of SNPs. **d** Genetic distances between groups measured as fixation indices (F_st_). The box colors depict the pairwise comparisons between groups. **e** Tajima’s *D*

To determine the evolutionary origin of the four *M. oryzae* groups identified in this study, we used a set of statistics that evaluate genetic diversity, recombination, and population differentiation. Initially, we visualized the relationships among samples using a phylogenetic network, which are more appropriate for visualizing reticulate evolution (Fig. 3a) [36]. We found that group I exhibited a high degree of reticulation. In contrast, the phylogenetic network showed long internal branches with terminal star-shape phylogenetic configurations almost devoid of reticulations for the well-defined groups II, III, and IV (Fig. 3a). Such configurations are typical of expanding populations after genetic bottlenecks, driven, for instance, by clonal expansions [37]. We, therefore, queried whether genetic diversity levels and recombination rates support clonality in groups II, III, and IV. Two lines of evidence support clonality in these groups compared with the diverse group I: (i) reduced nucleotide diversity measured as pi (π) [38] (Fig. 3b) and (ii) lower detectable recombination events calculated using the four-gamete test [39] (Fig. 3c). The reduced levels of diversity in groups II, III, and IV in conjunction with their star-like phylogenies are tell-tale signs of populations that have experienced a strong reduction of diversity followed by a population expansion. Reductions in diversity followed by population expansion are typical of both demographic bottlenecks or founder effects (i.e., the establishment of a new population from a reduced number of individuals). Independent of the exact nature of the demographic processes and evolutionary forces that gave rise to the changes in population size, the diversity and phylogenetic patterns that we observed are mostly driven by the population expansion phase. To calculate a proxy for recombination, we used the four-gamete test, which puts a bound to the minimum number of recombination events in a sample [39]. Although it is known that this test underestimates recombination events, it is a simple and useful proxy for differences in recombination between populations. Our results showed that groups II, III, and IV have on average ~ 5-fold less recombination events than the diverse group I. In agreement with our analysis, metainformation obtained by Gladieux et al. and Zhong et al. [31, 32] showed that in almost all cases, only one mating type was present in groups II, III, and IV, whereas the two mating types were segregating in the diverse group I (Additional file 1: Fig. S2 and Additional file 2: Table S1). In sum, we conclude that groups II, III, and IV are likely clonal lineages, while group I consists of genetically diverse and recombining individuals (Fig. 3a–c). The original microsatellite-based study by Saleh et al. [35] reported a high level of genetic variability in group IV; however, both our analyses and the ones carried out by Gladieux et al. [31] supported the clonal nature of this group.

To further investigate the relationships and demographic history of *M. oryzae* groups, we measured population differentiation among groups and leveraged the site frequency spectrum (SFS) for each group individually. To measure population differentiation, we used F_ST_ [40] and found that when clonal groups II, III, and IV are compared among them, their F_ST_ distances were the highest. Although a fraction of the allele frequency differences that resulted in high F_ST_ values could have been driven by selection, the fact that on average F_ST_ values are much higher among clonal groups likely reflects a long history of independent drift. In contrast, whenever the diverse group I is compared with any of the clonal groups, the F_ST_ distances decreased, suggesting that group I is a common source of genetic diversity for all clonal lineages (Fig. 3d). Subsequently, for every group, we investigated their corresponding SFS using Tajima’s *D* [41], as this statistic records changes in allele frequencies driven, for instance, by variation in population sizes. We found that Tajima’s *D* values for all clonal lineages were negative (Fig. 3e). A demographic interpretation of negative Tajima’s *D* values is consistent with population bottlenecks or founder effects followed by population expansions and a concurrent accumulation of rare alleles. Negative Tajima’s *D* values are consistent with star-like phylogenies, as new mutations that occurred during the expansion phase accumulate in terminal branches lowering Tajima’s *D* values. The inspection of the SFS also revealed an excess of high-frequency derived alleles, a feature of the SFS found mostly in rapidly adaptive populations, and that is particularly strong in asexual organisms or in organisms where meiotic recombination happen infrequently [42] (Additional file 1: Fig. S3). By using multiple outgroups, we discarded that our observation is caused by misassignemnt of the ancestral allele. We believe, instead, that the excess of high-frequency-derived alleles might be driven by a process dubbed genetic draft, i.e., the random association of alleles with genetic backgrounds of different fitness (Gillespie, 2000). Thus, although the SFS is mainly driven by genetic drift during the population expansion phase—as manifested by the negative Tajima’s *D*—linked selection via genetic draft contributes to the fate of neutral alleles. Further theoretical work is needed to quantify the role of genetic draft in clonal populations of *M. oryzae*.

Overall, our results are consistent with a model where Southeast Asia is a likely center of origin of rice-infecting *M. oryzae* and in which three distinct clonal lineages arose from this ancestral population. These findings are consistent with previous models of the evolution of the rice lineage of *M. oryzae* [35].

### *Magnaporthe oryzae* rice-infecting clonal lineages are estimated to have arisen in the last 200 years

**Fig. 4.**
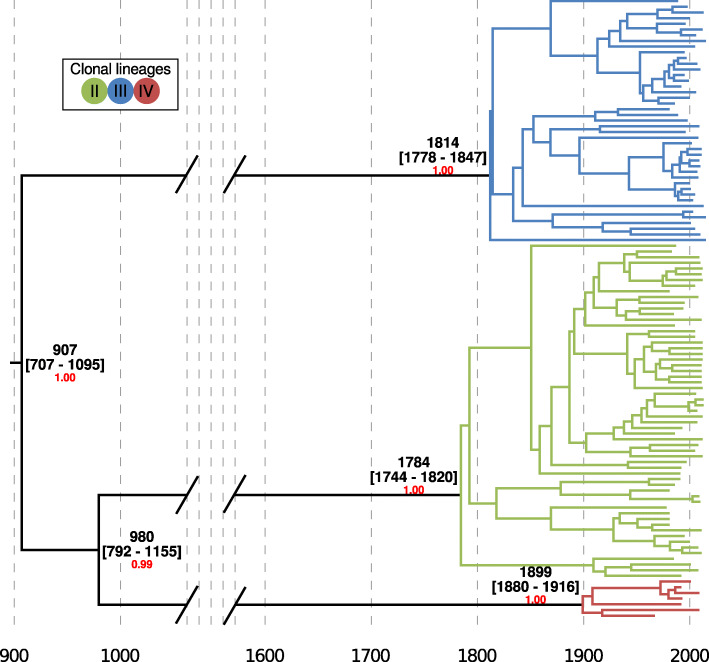
Clonal expansions of *Magnaporthe oryzae* took place in the last 200 years. Bayesian tip calibrated phylogenetic tree using individuals belonging to clonal lineages. Average, and HPD 95% confidence intervals are shown in calendar years. The Bayesian posterior probability is shown in red for nodes leading to the clonal lineage expansions

**Fig. 5.**
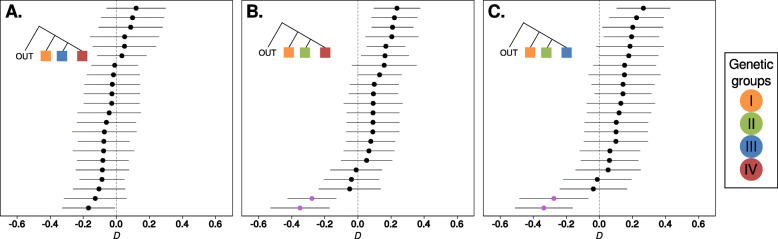
Patterns of allele frequency sharing identify introgression between a Chinese *Magnaporthe oryzae* subpopulation and clonal lineage II. *D*-statistics using three different phylogenetic configurations (depicted as colored inset trees). **a***D* (outgroup, orange; blue, red). **b***D* (outgroup, orange; green, red). **c***D* (outgroup, orange; green, blue). In all cases, a *M. oryzae* strain from wheat was used as an outgroup and a fixed individual was selected as representative from each clonal lineage (blue, orange, red). Points represent *D*-statistic tests for each of the 22 individuals assigned to the diverse clade (orange), and lines depict 95% confidence intervals. Purple dots in **b** and **c** correspond to Chinese individuals *CH1016* and *HB-LTH18*, which are the closest individuals to the green clonal lineage

To estimate the divergence time of the clonal expansions of *M. oryzae*, we first used a Bayesian phylogenetic analysis leveraging the sample collection dates for tip-calibration [43, 44]. To carry out the analysis, we first removed the diverse group I and used only the three clonal lineages, as the recombining group violates the assumptions of phylogenetic reconstruction. We used a concatenation approach including SNPs in the input pseudo-alignment. We also codified the amount of invariant sites in the configuration file, since the exclusion of invariant sites will lead to deeper divergence times (Additional file 1: Fig. S6). We estimated an evolutionary rate of 2.16e−8 substitutions/site/year (1.80e−8 − 2.55e−8 HPD 95%), which was similar and contains in its HPD 95% a previously calculated rate (1.98e−8 substitutions/site/year) [31]. Our approach of including only the clonal lineages permitted the reconstruction of a robust phylogeny and a more accurate estimation of divergence times, as reflected in the high posterior probabilities supporting the nodes and the narrow HPD 95% confidence intervals of node ages (Fig. 4). The topology of the tree—clearly separating all clonal lineages—and the divergence time estimates were robust when we tested the effect of the small sample size of clonal group IV (Additional file 1: Fig. S7). This contrasts with previous studies that included individuals from the diverse recombining group I in the phylogenetic analysis and produced broader HPD 95% confidence intervals (Fig. 5 [31]).

The phylogenetic reconstructions revealed that all three clonal expansions occurred relatively recently over the last 200 years (123–242) (Fig. 4). These expansions happened concomitantly with an increase of the effective population size of all clonal lineages (Additional file 1: Fig. S4).

To assess the robustness of the phylogenetic reconstruction, we carried out two additional analyses. First, we used a full phylogenetic method that takes into account incomplete lineage sorting. Instead of sampling all possible gene trees, the method computes a tree directly from the markers integrating over all possible gene trees [45] (Additional file 1: Fig. S8). Additionally, we used a coalescent-based method for multi-locus unlinked data that infers the quartet trees for all subsets of isolates and then combines the quartets in a single tree [46, 47] (Additional file 1: Fig. S9). Both analyses confirmed the monophyly of each clonal group.

### Patterns of allele frequency sharing identify introgression between a subpopulation of the diverse group I and clonal lineage II

Since the identification of admixture between populations facilitates the reconstruction of the evolutionary history of populations, we investigated the admixture history of *M. oryzae* using *D*-statistics [48, 49]. This test employs counts of site patterns, which are patterns of alternative alleles at a given genomic position, and evaluates whether these site patterns support one of two alternative discordant topologies. The *D*-statistics will return a value of zero if the two discordant phylogenies are supported equally, whereas positive or negative values indicate asymmetric support and, therefore, introgression. We test the three possible configurations of the following form: *D* (outgroup, diverse group I; clonal lineage X, clonal lineage Y) (tree insets in Fig. 5a–c). While for clonal lineages II, III, and VI, we used a strain representative for each clonal lineage, we performed a test for every one of the 22 members of the diverse group I. The test will retrieve positive values when the diverse group I is closer to clonal lineage Y and negative values when the diverse group I is closer to clonal lineage X. We found that group II has drifted farther apart from the diverse group I than the two other clonal lineages, as manifested from positive *D*-statistics when group II was included (as clonal lineage X) in the comparisons (Fig. 5b, c). This accumulation of genetic drift is consistent with the fact that group II was the clonal lineage that diverged earliest from the recombining diverse group (Fig. 4). We retrieved positive *D-*statistics in tests including almost all individuals of the diverse group I, with the exception of two individuals collected in China—*CH1016* [31] and *HB-LTH18* [32]—that showed strong signals of genetic introgression with the clonal lineage II, as manifested by negative *D*-statistic values (Fig. 5b, c). Since we detected introgression between these two Chinese samples and all members of group II regardless of their geographic origin (Additional file 1: Fig. S10A-B), we inferred that the admixture should have taken place before the clonal expansion that gave rise to group II about 197–294 years ago (Fig. 4). Previous attempts to detect interlineage recombination were not statistically robust and plagued with false positives [31]. In contrast, *D*-statistics provide a statistically robust framework that reliably permits distinguishing between introgression and incomplete lineage sorting using genome-wide SNPs [48, 49].

To further investigate the extent and location of the introgression between group II and the two Chinese Group I individuals (*CH1016/HB-LTH18*), we segmented the genomes of the two Chinese individuals based on their similarity at segregating sites to either group I or group II (Additional file 1: Fig. S11B). This analysis revealed that the genome-wide percentage of group II-like fragments in the Chinese individuals is 44.58%, including a ~ 4 Mb region in chromosome 3 (Additional file 1: Fig. S11B). To test whether those fragments are a good proxy for the percentage of introgression, we carried out two additional tests. First, we repeated the *D*-statistic test presented in Fig. 5b and supplementary Fig. 11A, but this time, removing the candidate introgressed fragments. In contrast to the outcome of the test with whole-genome data, this time the test did not indicate introgression, i.e., it was not different from zero (Additional file 1: Fig. S11C). Second, we estimated the proportion of introgression by using a f4-ratio test [50] with the following setup: (group III, group II, group I (without introgressed Chinese individuals), outgroup)/(group III, group II, Chinese introgressed individuals, outgroup). This test estimated the mixture proportion to be ~ 31.68%, a lower but similar value to the overall percentage of identified group II-like fragments in the Chinese individuals.

### Lineages of *Magnaporthe oryzae* show distinct patterns of presence and absence of effector genes

**Fig. 6.**
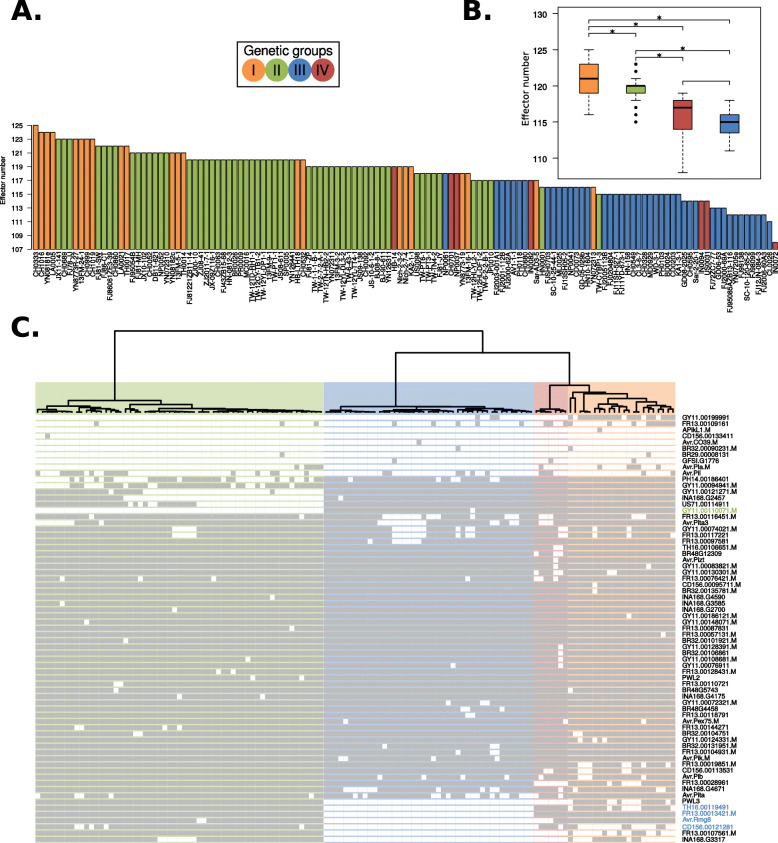
Rice blast genetic lineages vary in the number and patterns of presence and absence of candidate effector genes. **a** Clonal lineages carry a reduced repertoire of effector genes compared with the diverse group I. **b** The box-and-whisker plots show the distribution of effector number per isolate for each genetic group. Asterisks represent a *p* value < 0.01 for a one-tailed Wilcoxon non-parametric test. **c** The dendrogram shows the clustering based on f3-outgroup statistic (as in Fig. 1). Light and dark colors on the rows represent absence and presence of effectors, respectively. Rows were grouped using a hierarchical clustering algorithm. Labels in green and blue font denote effectors missing in clonal groups II and III, respectively

In *M. oryzae*, regions of the genome containing effector genes exhibit a high rate of structural variation as illustrated by presence and absence polymorphisms [25]. We investigated the distribution of known and predicted effector genes within the population structure framework we defined for the rice lineage of *M. oryzae*. We mapped the genome sequences of the 131 isolates to the sequences of 178 known and candidate effectors predicted from the genomes of *M. oryzae* from hosts as diverse as rice, wheat, finger millet, foxtail millet, oat, and *Digitaria* spp. [51]. This pan-effectorome set enabled us to capture as much effector gene diversity as possible. In total, 134 effectors were identified in the 131 isolates (Additional file 4: Table S3). Remarkably, the number of effectors per isolate varied from 108 to 125 with clonal lineages carrying a reduced repertoire of effector genes compared with the diverse genetic group (Fig. 6a, b). This indicates that clonal-expansion-driven bottlenecks not only reduced the overall genetic diversity of all pandemic clonal lineages but are associated with a less diverse repertoire of dispensable genes such as effectors. In pathogenic bacteria, a reduction in the effectiveness of purifying selection has been associated with an increase in gene loss [52]. Moreover, gene loss is particularly prevalent in clonal pathogenic bacteria and has been postulated as a source of phenotypic variation in these otherwise genetically similar species [53]. The association between gene loss and reduced purifying selection in bacteria is a consequence of their strong deletional bias, i.e., bacteria with reduced effective population size experience genome reduction [54]. In contrast, eukaryotes with small effective population sizes have larger genomes [55] and filamentous plant pathogens are notorious for having repeat-driven genome expansions associated with a “two-speed” architecture [56, 57]. This relation is, however, more complex in the rice blast fungal phylum Ascomycota, where both genome expansions and reductions have been observed [58]. It remains to be tested if the concurrent loss of genetic diversity and dispensable/non-core genes is a widespread consequence of clonality-driven bottlenecks, or if clonal expansions are driven by (adaptive) phenotypic novelty resulting from gene loss.

We next mapped the distribution of the subset of 69 effectors that display presence and absence polymorphisms across all strains (Fig. 6c). The resulting matrix clearly shows that there are distinct patterns of presence and absence of effectors across the genetically defined groups. For example, a set of four effectors (Avr-Rmg8, FR13.00013421, TH16.00119491, and CD156.00121281) are absent in group III. Likewise, PWL3, INA168.g3317, and FR13.00107561.M are absent in groups III and IV, GY11.00110071.M is absent in group II, and FR13.00028961 is absent in group IV (Fig. 6c, Additional file 5: Table S4).

To determine which effectors have the strongest association with the defined genetic structure, we conducted two separate analyses based on the presence and absence effector repertoire per isolate. First, a PCA and effector loadings analysis revealed a set of 13 effectors that explained 90% of the variance of both PC1 and PC2 (Additional file 1: Fig. S12A-B). Similarly, by using extremely randomized trees (a classification machine learning technique), we identified a set of 16 effectors that explained 90% of the variance (Additional file 1: Fig. S12C). Although the two methods produced different rankings of the impact of each effector gene, we found an overlap of 92.3% between the top 13 effectors found in the two subsets. In both cases, the top effectors reproduced the separation of the isolates in the described genetic clusters (Additional file 1: Fig. S12D-E). A close inspection of this group of top effectors, which were selected in an unbiased way, revealed that they are differentially (almost) present or (almost) absent in the four *M. oryzae* genetic groups (Additional file 5: Table S4). Thus, this group of effectors might have played an important role in the initial adaptation of *M. oryzae* clonal expansions to different rice subspecies and varieties.

The matrix in Fig. 6c indicates that patterns of presence and absence of effector genes reflect different timescales in the evolution of the clonal lineages of *M. oryzae*. AVR effectors, such as AVR-Pia and AVR-Pii, show a patchy distribution within the clonal lineages. Their recurrent deletion in *M. oryzae* populations has generated virulent races [27]. This may reflect the fact that their matching resistance genes have been repeatedly bred and deployed into rice cultivars. Other candidate effectors that display a similar patchy distribution may be candidate AVR effectors that are detected by one of the dozens of blast resistance genes that have been bred into rice cultivars.

Our finding that the clonal lineages of rice-infecting *M. oryzae* display distinct repertoires of effectors raises a number of interesting questions. It is possible that this reflects the distinct genotype of the founding individual of the given clone. It is also possible that the absence of a given AVR effector(s) has facilitated the spread of the clonal lineage to otherwise resistant host genotypes as previously noted in *M. oryzae* [22, 25, 30, 59]. In the future, it would be interesting to test the extent to which effectors that define the clonal lineages are detected by particular resistance genes. For example, AVR-Rmg8, which is known in wheat blast isolates to mediate avirulence on Rmg8 containing wheat varieties, may also be detected by a rice resistance gene. Future experiments will tease out the degree to which the distinct effector repertoires of the clonal lineages of *M. oryzae* reflect their adaptation to the rice host and their evolutionary history. Such analyses will require new genomic resources that permit a more accurate identification of effectors in canonical chromosomes and mini-chromosomes [60, 61]. To this aim, it will be fundamental to generate multiple reference genomes sequenced with long-read technologies in conjunction with a detailed characterization of structural variation and genomic rearrangements, which will include a per isolate inventory of mini-chromosome repertoires.

## Conclusion

Our analyses reconstruct the genetic history of the rice-infecting lineage of *M. oryzae* revealing three clonal lineages that have emerged over the last ~ 100–200 years and have been associated with rice blast pandemics. These lineages display differential loss of effector genes that may have shaped their adaptation to the rice host and their evolutionary history. These findings provide a framework for further comparative analyses of the genomes of rice-infecting *M. oryzae*. One particular interesting research avenue will be to establish the degree to which structural variation, notably mini-chromosomes, has impacted the evolution of this lineage.

## Methods

### Datasets and mapping

We used *M. oryzae* Illumina reads from two recent resequencing studies (43 samples from Gladieux et al. [31], and 88 samples from Zhong et al. [32] (Additional file 2: Table S1)). Raw sequencing reads were downloaded and mapped to the *M. oryzae* reference genome (*GUY-11* PacBio assembly [62]) using *bwa-mem* V.0.7.12 [63] with default parameters.

### Variant identification and filtering

De novo variants were identified using *GATK* V.3.8.0 [64]. The following set of filters were applied: QD < 5.0; QUAL < 5000.0; MQ < 20.0; − 2.0 < ReadPosRankSum < 2.0; − 2.0 < MQRankSum < 2.0; − 2.0 < BaseQRankSum < 2.0. In all subsequent analyses, we used only biallelic SNPs present in all samples (“full information”).

### Population structure analyses

To assess the global population structure of *M. oryzae*, we first determined patterns of allele sharing using f3-outgroup statistics [33]. We performed the test using the program *qp3Pop* from the *AdmixTools* package [50]. The test was used to establish the pairwise relatedness between *M. oryzae* samples (X and Y) after divergence from an outgroup: *f3*(X, Y; outgroup). We used a deeply diverged *Setaria*-infecting *M. oryzae* strain SA05-144 [25] as outgroup. We calculated *z*-scores for every possible pairwise sample comparison included in the f3-statistics test (*N* = 8515). Subsequently, we carried out hierarchical clustering using the function *hclust* from the *R* package *stats* [65]. As input, we used a distance matrix generated from the f3-statistics-derived *z*-scores (Fig. 1a).

Additionally, we determined the level of population structure using genetic distances coupled with dimensionality reduction methods. We calculated pairwise Hamming distances using *Plink V.1.9* [66]. Such distances were used as input for principal component analysis (PCA) using the function *prcomp* from the *R* package *stats* [65] (Additional file 1: Fig. S1A). To assess the robustness of the clusters, PCA coordinates were used to compute silhouette scores using the function *silhouette* from the *R* package *cluster* [67]. We calculated mean silhouette scores for different numbers of clusters (*K* = 2–6) and found that the highest mean silhouette scores were obtained when *K* = 4. We also used Discriminant Analysis of Principal Components (DAPC) [68], implemented in the *adegenet R* package. The analysis was carried out by capturing the variance in the 10 first PC’s. The Bayesian information criterion (BIC) indicated that the best number of groups was *K* = 4 (Additional file 1: Fig. S1C-D). We used the grouping of individuals in four clusters for subsequent analyses.

### Population genetics analyses

We constructed a neighbor network using the program *SplitsTree V.4.14.6* [36]. As a proxy for recombination within each of the clusters, we used the four-gamete test [39] as implemented in *RminCutter* [69]. To this aim, we created consensus *fasta* sequences from the contigs 1 to 7 using the filtered vcf file with *bcftools* V. 1.3.1 [70]. The summary statistic was calculated by dividing the total number of violation events of the four-gamete test by the total number of SNPs. Nucleotide diversity (π), fixation indices F_ST_, and Tajima’s *D* values were calculated using *vcftools V.0.5.15* [71]. We calculated the unfolded site spectrum (SFS) for each genetic group using custom scripts. Ancestral alleles were ascertained requiring concordance between a *Setaria*- and a wheat-infecting outgroup strain (SA05-144 [25] and BTJP-4(12) [72]).

We computed *D*-statistic values [48] as follows:

$$ D\left(O,T;X,Y\right)=\frac{\left({p}_O-{p}_T\right)\ \left({p}_X-{p}_Y\right)}{\left({p}_O+{p}_T-2{p}_O{p}_T\right)\ \left({p}_X+{p}_Y-2{p}_X{p}_Y\right)} $$

where *P*_*O*_, *P*_*T*_, *P*_*X*_, and *P*_*Y*_ are frequencies of randomly selected alleles in populations (*O*)utgroup, (*T*)est, *X*, and *Y* at each locus. The reported 95% confidence intervals were calculated as *D* ± (SE × 1.96) where the standard error was computed using a jackknife weighted by the number of SNPs for each 5 Mb block in the genome [73]. We performed the calculations using *popstats* [74].

### Genomic segmentation analysis

Based on the *D*-statistic results, two isolates from the diverse group I (CH1016 and HB-LTH18) showed genome-wide introgression evidence with the clonal lineage II. In order to identify which regions of the genomes of CH1016 and HB-LTH18 show higher nucleotide similarity to clonal lineage II than to members of the diverse group I, we performed a window-based similarity analysis. These regions, especially if they overlap between CH1016 and HB-LTH18, will be strong candidates for being introgressed from the clonal lineage II. Consequently, we performed window-based pairwise nucleotide similarity comparisons between an example isolate of clonal lineage II (TW-PT3-1) and the two Chinese individuals (CH1016 and HB-LTH18). To this end, we divided the seven chromosomes in 400 windows, each of which had the same number of SNPs. To ascertain the basal level of similarity among clonal lineage II individuals, we compared our example clonal lineage II isolate TW-PT3-1 with another clonal lineage II isolate (BR0026). Finally, to ascertain the nucleotide similarity between clonal lineage II and non-introgressed individuals from the diverse group I, we compared our example clonal lineage II isolate TW-PT3-1 with diverse group I isolates CH0532 and CH0333.

### Phylogenetic analysis

We first carried out a Bayesian tip-dated phylogenetic analysis. To perform this analysis, we first removed individuals from the diverse group I, as these recombining group of individuals do not comply with the assumptions of any phylogenetic analysis (Fig. 2c). We kept only biallelic variant positions to perform a Markov chain Monte Carlo-based phylogenetic reconstruction using *BEAST V.2.4.8* [75]. We used the isolates’ collection dates (Additional file 2: Table S1) as prior information for the estimation of divergence times. We used *ModelTest-NG* [76] to assess the best suitable substitution model. Based on the lowest Akaike information criterion (AIC), we selected the general time-reversible model. Since the calculation was performed with non-recombining individuals from the same species, we used a strict clock rate with a prior value of 1.98e−8 substitutions/site/year, which was the rate ascertained in Gladieux et al. [31]. To test the hypothesis of a non-clocklike data, we estimated the coefficient of variation in a model relaxed clock log normal model to be 0.0042, suggesting strong evidence for a clock-like data [77]. In order to reduce the effect of demographic history assumptions, and to calculate the dynamics of the population size through time, we also chose a Coalescent Extended Bayesian Skyline approach [78]. Invariant sites were explicitly considered in the model by adding a “*constantSiteWeights*” tag in the XML configuration file. We combined the output of four independent MCMC chains. Each chain had a length of 10 million iterations and was logged every 1000 iterations. We only used chains with overall ESS values above 200 and summarized a maximum clade credibility tree with *TreeAnnotator*. We summarized effective population size through time using an Extended Bayesian Skyline Plot (Additional file 1: Fig. S4). Configuration and log files are provided in our repository (see code and data availability).

To assess the robustness of the phylogenetic reconstruction to different sources of tree dicordances, we carried out several additional analyses. First, to illustrate the effect of recombination in the phylogeny, we included all the individuals from the genetic group I, who displayed signatures of sexual recombination (Fig. 2c) and computed a new phylogeny following the same approach employed for the clonal groups (Additional file 1: Fig. S5). To evaluate the effect of unequal sample sizes on the topology and divergence times of the clonal groups, we downsampled both genetic groups II and III to *N* = 7 (the number of isolates from the genetic group IV). Subsequently, we repeated the phylogenetic used originally for the clonal groups. (Additional file 1: Fig. S7). As an alternative to our described concatenation approach, we implemented two full phylogenetic methods that take into account incomplete lineage sorting and assume free recombination between variant sites. First, making use of the genomic SNP dataset, we utilized the SNAPP implementation provided for BEAST2 [45]. We assumed no prior tree and mutation rate values *U* and *V* were set to their defaults (1.0). We set the MCMC chain length of 10 million iterations with sampling every 1000 iterations (Additional file 1: Fig. S8). Configuration and log files are provided in our repository (see code and data availability). Finally, we also used SVDquartets [46, 47] with its implementation in PAUP V4.0a [79].. We selected a multispecies coalescent tree with an exhaustive examination of all possible quartets (*N* = 5,563,251). To assess node support, a bootstrap with 100 replicates was implemented (Additional file 1: Fig. S9).

### Effector genes repertoire

To determine the effector gene repertoire for each of 131 *M. oryzae* isolates described in Additional file 2: Table S1, we mapped the publicly available genomic short-read sequences from these isolates to a reference set of diverse effector candidate sequences. We used the recently reported database from Petit-Houdenot and colleagues [51], which is composed by 195 candidates with similarity to both AVR and MAX effectors from isolates infecting a wide variety of hosts (e.g., rice, wheat, finger millet, foxtail millet, oat, and other *Digitaria* species). We reduced the redundancy of the reference by removing highly similar sequences (≥ 90% identity). The final reference set included 178 coding DNA sequences for candidate effectors (Additional file 6: Table S5) from different *M. oryzae* lineages infecting hosts such as rice, wheat, oat, millet, and wild grasses. The coordinates of the reference effector genes corresponding to *M. oryzae* PacBio genome GUY-11 (GenBank accession GCA_002368485.1) are shown in Supplementary Table 4. We used elongation factor 2 mRNA sequence (GenBank accession XM_003714691.1) from *M. oryzae* as a positive control for presence of a gene, and a secreted protein gene *CoMC69* from the fungus *Colletotrichum orbiculare* as a negative control for absence of a gene in the reference for short-read mapping.

Mapping was performed with *bwa-mem* V.0.7.15 [80]. An effector was deemed present if more than 80% of its sequence was recovered with a minimum depth of 3x, using *SAMtools* V1.6 [81].

To summarize effector content per isolate, we built a presence and absence matrix indicating presence and absence of effector genes with 1 and 0, respectively (Fig. 6a, b and Additional file 7: Table S6). For subsequent analyses, we excluded effector genes that were either present or absent in all lineages, as they are uninformative for clustering algorithms. This filtering resulted in a presence and absence matrix that contains a set of 69 informative effectors. We organized the columns of this matrix according to the dendrogram of genetic groups (Fig. 1), while the rows were sorted using hierarchical clustering with the function *hclust* from *R stats* package [65].

To determine which effectors have the strongest association with the defined genetic structure of *M. oryzae*, we conducted PCA and loading analysis using the presence and absence matrix per isolate as input. Analyses were carried out with the *princomp* function of the *R stats* package [65]. Then, by multiplying the absolute value of X and Y coordinates of each loading vector in PC1 and PC2, we assessed the strength of importance per effector (Additional file 1: Fig. S12A). We selected a subset of effectors that contains the 13 most important effectors (i.e., loading vectors with the highest magnitudes), as they together explained 90% of the variance (Additional file 1: Fig. S12C). We recalculated the PCA using only this subset of 13 effectors, which resulted in an increase from 44.8 to 73.8% (total increase of 29%) of the variance explained by PC1 and PC2 together.

A similar analysis was also carried out using the extremely randomized trees algorithm implemented in the *Python scikit-learn* module [82], with 100 trees per forest, and trained using all the effector presence and absence data. The feature importances were extracted from the trained model. This process was repeated 2500 times to ensure consistency, and the mean effector importance for reconstructing the population structure was calculated in order to rank the effectors. Using this method, 90% of the variance was explained by 16 effectors (Additional file 1: Fig. S12C).

## Supplementary information

**Additional file 1: Fig. S1**. Principal component analysis (PCA) reveals four defined groups. **Fig. S2**. Relation between genetic groups and sample mating type**. Fig. S3**. The unfolded Site Frequency Spectrum (SFS). **Fig. S4**. Recent increase of population size in clonal lineages of Magnaporthe oryzae. **Fig. S5**. Effect of recombination on the Magnaporthe oryzae phylogeny construction. **Fig. S6**. Estimated Time to Most Recent Common Ancestor (TMRCA) for clonal lineages with / without invariant sites. **Fig. S7**. Effect of sample size on the topology of the tree and on the estimation of divergence times. **Fig. S8**. Phylogenetic inference using Single Nucleotide Polymorphisms using SNAPP. **Fig. S9**. Phylogenetic inference using SVDquartets. **Fig. S10**. Two Chinese individuals display consistent introgression with the clonal lineage II. **Fig. S11**. Ancestry-based genomic segmentation of Chinese individuals CH1016 and HB-LTH18 reveals a 4 Mb putative introgressed region on chromosome 3. **Fig. S12**. Effector loadings reveal major effector loss in clonal lineage III.

**Additional file 2: Table S1**. Information of isolates used in this study. (TSV 5 kb)

**Additional file 3: Table S2**. New classification assessed in this study. (TSV 2 kb)

**Additional file 4: Table S3**. Members of the pan-effectorome that are present or absent in all 131 *Magnaporthe oryzae* isolates used in this study along with the effectors showing presence and absence polymorphism.

**Additional file 5: Table S4**. Patterns of effector presence and absence polymorphism in *Magnaporthe oryzae* isolates

**Additional file 6: Table S5**. Effector nucleotide sequences. (TSV 71 kb)

**Additional file 7: Table S6**. Binary assessment of effectors per isolate. (TSV 49 kb)

## Data Availability

The datasets and scripts generated during and/or analyzed during the current study are available in the Gitlab repository, https://gitlab.com/smlatorreo/genetic_history_of_rice-infecting_magnaporthe_oryzae [83].

## References

[1] Fisher MC, Henk DA, Briggs CJ, Brownstein JS, Madoff LC, McCraw SL (2012). Emerging fungal threats to animal, plant and ecosystem health. Nature..

[2] Savary S, Willocquet L, Pethybridge SJ, Esker P, McRoberts N, Nelson A (2019). The global burden of pathogens and pests on major food crops. Nat Ecol Evol.

[3] Carvajal-Yepes M, Cardwell K, Nelson A, Garrett KA, Giovani B, Saunders DGO (2019). A global surveillance system for crop diseases. Science..

[4] Grandaubert J, Dutheil JY, Stukenbrock EH (2019). The genomic determinants of adaptive evolution in a fungal pathogen. Evol Lett.

[5] Croll D, Laine A-L (2016). What the population genetic structures of host and pathogen tell us about disease evolution. The New phytologist.

[6] Terauchi R, Yoshida K (2010). Towards population genomics of effector-effector target interactions: research review. New Phytol.

[7] Cooke DEL, Cano LM, Raffaele S, Bain RA, Cooke LR, Etherington GJ (2012). Genome analyses of an aggressive and invasive lineage of the Irish potato famine pathogen. PLoS Pathog.

[8] Islam MT, Croll D, Gladieux P, Soanes DM, Persoons A, Bhattacharjee P (2016). Emergence of wheat blast in Bangladesh was caused by a South American lineage of Magnaporthe oryzae. BMC Biol.

[9] Hubbard A, Lewis CM, Yoshida K, Ramirez-Gonzalez RH, de Vallavieille-Pope C, Thomas J (2015). Field pathogenomics reveals the emergence of a diverse wheat yellow rust population. Genome Biol.

[10] Radhakrishnan GV, Cook NM, Bueno-Sancho V, Lewis CM, Persoons A, Mitiku AD (2019). MARPLE, a point-of-care, strain-level disease diagnostics and surveillance tool for complex fungal pathogens. BMC Biol.

[11] Saunders DGO, Pretorius ZA, Hovmøller MS (2019). Tackling the re-emergence of wheat stem rust in Western Europe. Commun Biol.

[12] Goss EM, Larsen M, Chastagner GA, Givens DR, Grünwald NJ (2009). Population genetic analysis infers migration pathways of Phytophthora ramorum in US nurseries. PLoS Pathog.

[13] Mohd-Assaad N, McDonald BA, Croll D (2019). The emergence of the multi-species NIP1 effector in Rhynchosporium was accompanied by high rates of gene duplications and losses. Environ Microbiol.

[14] Vleeshouwers VGAA, Oliver RP (2014). Effectors as tools in disease resistance breeding against biotrophic, hemibiotrophic, and necrotrophic plant pathogens. Mol Plant-Microbe Interact.

[15] Vleeshouwers VGAA, Rietman H, Krenek P, Champouret N, Young C, Oh S-K (2008). Effector genomics accelerates discovery and functional profiling of potato disease resistance and phytophthora infestans avirulence genes. PLoS One.

[16] Rietman H, Bijsterbosch G, Cano LM, Lee H-R, Vossen JH, Jacobsen E (2012). Qualitative and quantitative late blight resistance in the potato cultivar Sarpo Mira is determined by the perception of five distinct RXLR effectors. Mol Plant-Microbe Interact.

[17] Bebber DP, Holmes T, Gurr SJ (2014). The global spread of crop pests and pathogens. Glob Ecol Biogeogr.

[18] Bebber DP, Gurr SJ (2015). Crop-destroying fungal and oomycete pathogens challenge food security. Fungal Genet Biol.

[19] Nations U, United Nations. World Population Prospects 2019: Highlights. Statistical Papers - United Nations (Ser. A), Population and Vital Statistics Report. 2019. doi:10.18356/13bf5476-en.

[20] Dean R, Van Kan JAL, Pretorius ZA, Hammond-Kosack KE, Di Pietro A, Spanu PD (2012). The top 10 fungal pathogens in molecular plant pathology. Mol Plant Pathol.

[21] Islam MT, Kim K-H, Choi J (2019). Wheat blast in Bangladesh: the current situation and future impacts. Plant Pathol J.

[22] Inoue Y, Vy TTP, Yoshida K, Asano H, Mitsuoka C, Asuke S (2017). Evolution of the wheat blast fungus through functional losses in a host specificity determinant. Science..

[23] Saleh D, Xu P, Shen Y, Li C, Adreit H, Milazzo J (2012). Sex at the origin: an Asian population of the rice blast fungus Magnaporthe oryzae reproduces sexually. Mol Ecol.

[24] Chiapello H, Mallet L, Guérin C, Aguileta G, Amselem J, Kroj T (2015). Deciphering genome content and evolutionary relationships of isolates from the fungus Magnaporthe oryzae attacking different host plants. Genome Biol Evol.

[25] Yoshida K, Saunders DGO, Mitsuoka C, Natsume S, Kosugi S, Saitoh H (2016). Host specialization of the blast fungus Magnaporthe oryzae is associated with dynamic gain and loss of genes linked to transposable elements. BMC Genomics.

[26] Gladieux P, Condon B, Ravel S, Soanes D, Maciel JLN, Nhani A Jr, et al. Gene flow between divergent cereal- and grass-specific lineages of the rice blast fungus Magnaporthe oryzae. MBio. 2018;9. 10.1128/mBio.01219-17. 10.1128/mBio.01219-17 PMC5829825 29487238

[27] Yoshida K, Saitoh H, Fujisawa S, Kanzaki H, Matsumura H, Yoshida K (2009). Association genetics reveals three novel avirulence genes from the rice blast fungal pathogen Magnaporthe oryzae. Plant Cell.

[28] Białas A, Zess EK, De la Concepcion JC, Franceschetti M, Pennington HG, Yoshida K (2018). Lessons in effector and NLR biology of plant-microbe systems. Mol Plant-Microbe Interact.

[29] Dean RA, Talbot NJ, Ebbole DJ, Farman ML, Mitchell TK, Orbach MJ (2005). The genome sequence of the rice blast fungus Magnaporthe grisea. Nature..

[30] Xue M, Yang J, Li Z, Hu S, Yao N, Dean RA (2012). Comparative analysis of the genomes of two field isolates of the rice blast fungus Magnaporthe oryzae. PLoS Genet.

[31] Gladieux P, Ravel S, Rieux A, Cros-Arteil S, Adreit H, Milazzo J, et al. Coexistence of multiple endemic and pandemic lineages of the rice blast pathogen. MBio. 2018;9. 10.1128/mBio.01806-17. 10.1128/mBio.01806-17 PMC5885030 29615506

[32] Zhong Z, Chen M, Lin L, Han Y, Bao J, Tang W, et al. Population genomic analysis of the rice blast fungus reveals specific events associated with expansion of three main clades. ISME J. 2018. 10.1038/s41396-018-0100-6. 10.1038/s41396-018-0100-6 PMC6051997 29568114

[33] Raghavan M, Skoglund P, Graf KE, Metspalu M, Albrechtsen A, Moltke I (2014). Upper Palaeolithic Siberian genome reveals dual ancestry of Native Americans. Nature..

[34] Lovmar L, Ahlford A, Jonsson M, Syvänen A-C (2005). Silhouette scores for assessment of SNP genotype clusters. BMC Genomics.

[35] Saleh D, Milazzo J, Adreit H, Fournier E, Tharreau D (2014). South-East Asia is the center of origin, diversity and dispersion of the rice blast fungus. Magnaporthe oryzae New Phytol.

[36] Huson DH, Bryant D (2006). Application of phylogenetic networks in evolutionary studies. Mol Biol Evol.

[37] Exposito-Alonso M, Becker C, Schuenemann VJ, Reiter E, Setzer C, Slovak R (2018). The rate and potential relevance of new mutations in a colonizing plant lineage. PLoS Genet.

[38] Nei M, Li WH (1979). Mathematical model for studying genetic variation in terms of restriction endonucleases. Proc Natl Acad Sci U S A.

[39] Hudson RR, Kaplan NL (1985). Statistical properties of the number of recombination events in the history of a sample of DNA sequences. Genetics..

[40] Weir BS, Cockerham CC (1984). Estimating F-statistics for the analysis of population structure. Evolution..

[41] Tajima F (1989). Statistical method for testing the neutral mutation hypothesis by DNA polymorphism. Genetics..

[42] Neher RA (2013). Genetic draft, selective interference, and population genetics of rapid adaptation. Annu Rev Ecol Evol Syst.

[43] Drummond AJ, Bouckaert RR. Bayesian Evolutionary Analysis with BEAST. Cambridge University Press; 2015.

[44] Heled J, Drummond AJ (2012). Calibrated tree priors for relaxed phylogenetics and divergence time estimation. Syst Biol.

[45] Bryant D, Bouckaert R, Felsenstein J, Rosenberg NA, RoyChoudhury A (2012). Inferring species trees directly from biallelic genetic markers: bypassing gene trees in a full coalescent analysis. Mol Biol Evol.

[46] Chifman J, Kubatko L (2014). Quartet inference from SNP data under the coalescent model. Bioinformatics..

[47] Chifman J, Kubatko L (2015). Identifiability of the unrooted species tree topology under the coalescent model with time-reversible substitution processes, site-specific rate variation, and invariable sites. J Theor Biol.

[48] Green RE, Krause J, Briggs AW, Maricic T, Stenzel U, Kircher M (2010). A draft sequence of the Neandertal genome. Science..

[49] Durand EY, Patterson N, Reich D, Slatkin M (2011). Testing for ancient admixture between closely related populations. Mol Biol Evol.

[50] Patterson N, Moorjani P, Luo Y, Mallick S, Rohland N, Zhan Y (2012). Ancient admixture in human history. Genetics..

[51] Petit-Houdenot Y, Langner T, Harant A, Win J, Kamoun S. A clone resource of Magnaporthe oryzae effectors that share sequence and structural similarities across host-specific lineages 2019. doi:10.5281/zenodo.3268775. 10.1094/MPMI-03-20-0052-A 32460610

[52] Hershberg R, Tang H, Petrov DA (2007). Reduced selection leads to accelerated gene loss in Shigella. Genome Biol.

[53] Bolotin E, Hershberg R (2015). Gene loss dominates as a source of genetic variation within clonal pathogenic bacterial species. Genome Biol Evol..

[54] Mira A, Ochman H, Moran NA (2001). Deletional bias and the evolution of bacterial genomes. Trends Genet.

[55] Lynch M, Conery JS (2003). The origins of genome complexity. Science..

[56] Raffaele S, Kamoun S (2012). Genome evolution in filamentous plant pathogens: why bigger can be better. Nat Rev Microbiol.

[57] Dong S, Raffaele S, Kamoun S (2015). The two-speed genomes of filamentous pathogens: waltz with plants. Curr Opin Genet Dev.

[58] Kelkar YD, Ochman H (2012). Causes and consequences of genome expansion in fungi. Genome Biol Evol..

[59] Huang J, Si W, Deng Q, Li P, Yang S (2014). Rapid evolution of avirulence genes in rice blast fungus Magnaporthe oryzae. BMC Genet.

[60] Peng Z, Oliveira-Garcia E, Lin G, Hu Y, Dalby M, Migeon P (2019). Effector gene reshuffling involves dispensable mini-chromosomes in the wheat blast fungus. PLoS Genet.

[61] Langner T, Harant A, Gomez-Luciano LB, Shrestha RK, Win J, Kamoun S. Genomic rearrangements generate hypervariable mini-chromosomes in host-specific lineages of the blast fungus. bioRxiv. 2020. doi:10.1101/2020.01.10.901983. 10.1371/journal.pgen.1009386 PMC7909708 33591993

[62] Bao J, Chen M, Zhong Z, Tang W, Lin L, Zhang X (2017). PacBio sequencing reveals transposable elements as a key contributor to genomic plasticity and virulence variation in Magnaporthe oryzae. Mol Plant.

[63] Li H, Durbin R (2009). Fast and accurate short read alignment with Burrows-Wheeler transform. Bioinformatics..

[64] McKenna A, Hanna M, Banks E, Sivachenko A, Cibulskis K, Kernytsky A (2010). The Genome Analysis Toolkit: a MapReduce framework for analyzing next-generation DNA sequencing data. Genome Res.

[65] R Core Team. R: A language and environment for statistical computing. 2018. https://www.R-project.org/..

[66] Purcell S, Neale B, Todd-Brown K, Thomas L, Ferreira MAR, Bender D (2007). PLINK: a tool set for whole-genome association and population-based linkage analyses. Am J Hum Genet.

[67] Maechler M, Rousseeuw P, Struyf A, Hubert M, Hornik K. Cluster: cluster analysis basics and extensions 2012;1. http://dx.doi.org/. Accessed 4 Oct 2016.

[68] Jombart T, Devillard S, Balloux F (2010). Discriminant analysis of principal components: a new method for the analysis of genetically structured populations. BMC Genet.

[69] Ross-Ibarra J. RminCutter. GitHub repository. 2013. https://github.com/RILAB/rmin_cut.

[70] Bcftools by samtools. http://samtools.github.io/bcftools/. Accessed 30 Aug 2018.

[71] Danecek P, Auton A, Abecasis G, Albers CA, Banks E, DePristo MA (2011). The variant call format and VCFtools. Bioinformatics..

[72] Soanes D, Ryder LS, Islam MT, Talbot NJ (2017). Genome assemblies of Magnaporthe oryzae isolated from Bangladesh in 2016 and 2017.

[73] Reich D, Thangaraj K, Patterson N, Price AL, Singh L (2009). Reconstructing Indian population history. Nature..

[74] Skoglund P, Mallick S, Bortolini MC, Chennagiri N, Hünemeier T, Petzl-Erler ML (2015). Genetic evidence for two founding populations of the Americas. Nature..

[75] Bouckaert R, Heled J, Kühnert D, Vaughan T, Wu C-H, Xie D (2014). BEAST 2: a software platform for Bayesian evolutionary analysis. PLoS Comput Biol.

[76] Darriba D, Posada D, Stamatakis A. ModelTest-NG. Github. https://github.com/ddarriba/modeltest. Accessed 1 Aug 2019.

[77] Drummond AJ, Ho SYW, Phillips MJ, Rambaut A (2006). Relaxed phylogenetics and dating with confidence. PLoS Biol.

[78] Drummond AJ, Rambaut A, Shapiro B, Pybus OG (2005). Bayesian coalescent inference of past population dynamics from molecular sequences. Mol Biol Evol.

[79] Swofford DL (2002). PAUP: phylogenetic analysis using parsimony, version 4.0 b10.

[80] Li H. Aligning sequence reads, clone sequences and assembly contigs with BWA-MEM. arXiv [q-bio.GN]. 2013. http://arxiv.org/abs/1303.3997.

[81] Li H, Handsaker B, Wysoker A, Fennell T, Ruan J, Homer N (2009). The sequence alignment/map format and SAMtools. Bioinformatics..

[82] Pedregosa F, Varoquaux G, Gramfort A, Michel V, Thirion B, Grisel O, et al. Scikit-learn: Machine Learning in Python. J Mach Learn Res. 2011;12:2825–30.

[83] Latorre SM, Reyes-Avila CS, Malmgren A, Win J, Kamoun S, Burbano HA. Dataset and Scripts for: differential loss of effector genes in three recently expanded pandemic clonal lineages of the rice blast fungus 2020. doi:10.5281/zenodo.3893626. 10.1186/s12915-020-00818-z PMC7364606 32677941

