# Supplementary material for: Differential loss of effector genes in three recently expanded pandemic clonal lineages of the rice blast fungus
Source: BMC Biol. 2020 Jul 16;18:88. doi: 10.1186/s12915-020-00818-z (PMC7364606; doi:10.1186/s12915-020-00818-z)
Supplement: Supplementary file 1 — Additional file 1: Fig. S1. Principal component analysis (PCA) reveals four defined groups. Fig. S2. Relation between genetic groups and sample mating type. Fig. S3. The unfolded Site Frequency Spectrum (SFS). Fig. S4. Recent increase of population size in clonal lineages of Magnaporthe oryzae. Fig. S5. Effect of recombination on the Magnaporthe oryzae phylogeny construction. Fig. S6. Estimated Time to Most Recent Common Ancestor (TMRCA) for clonal lineages with / without invariant sites. Fig. S7. Effect of sample size on the topology of the tree and on the estimation of divergence times. Fig. S8. Phylogenetic inference using Single Nucleotide Polymorphisms using SNAPP. Fig. S9. Phylogenetic inference using SVDquartets. Fig. S10. Two Chinese individuals display consistent introgression with the clonal lineage II. Fig. S11. Ancestry-based genomic segmentation of Chinese individuals CH1016 and HB-LTH18 reveals a 4 Mb putative introgressed region on chromosome 3. Fig. S12. Effector loadings reveal major effector loss in clonal lineage III. [file 12915_2020_818_MOESM1_ESM.pdf]

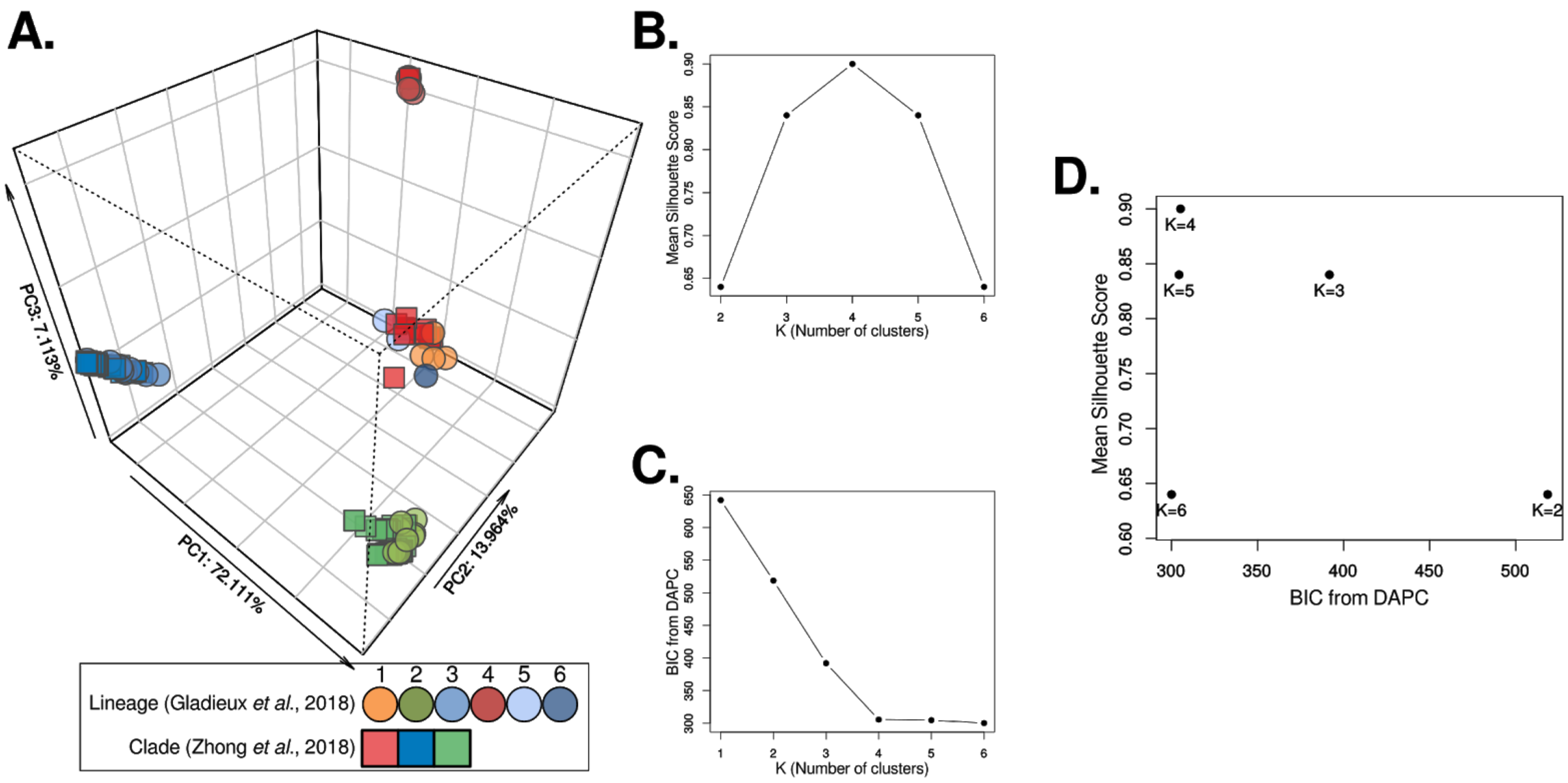

Figure S1. Principal component analysis (PCA) reveals four defined groups. **(A)** PCA based on pairwise Hamming distances. **(B)** Silhouette score analysis shows best averages per group scores when *K*=4. **(C)** Discriminant Analysis of Principal Components (DAPC) shows stabilization of the Bayesian Information Criterion (BIC) when *K*=4. **(D)** BIC from DAPC versus mean silhouette scores shows an optimal number of groups when *K*=4.

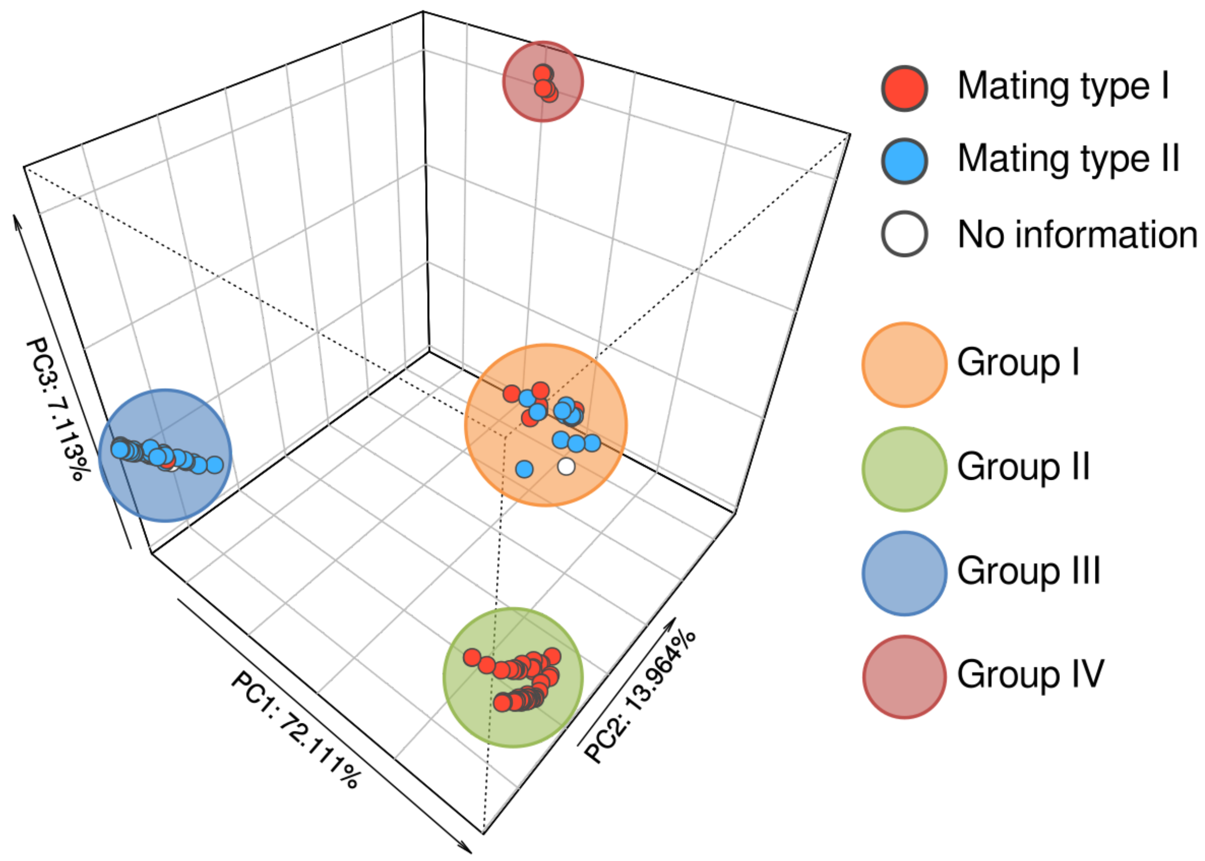

Figure S2. Relation between genetic groups and sample mating type. PCA based on pairwise Hamming distances (same coordinates as Additional file 1:Supp. Fig. S1). The color scheme codifies the mating type described as metainformation in the previous studies.

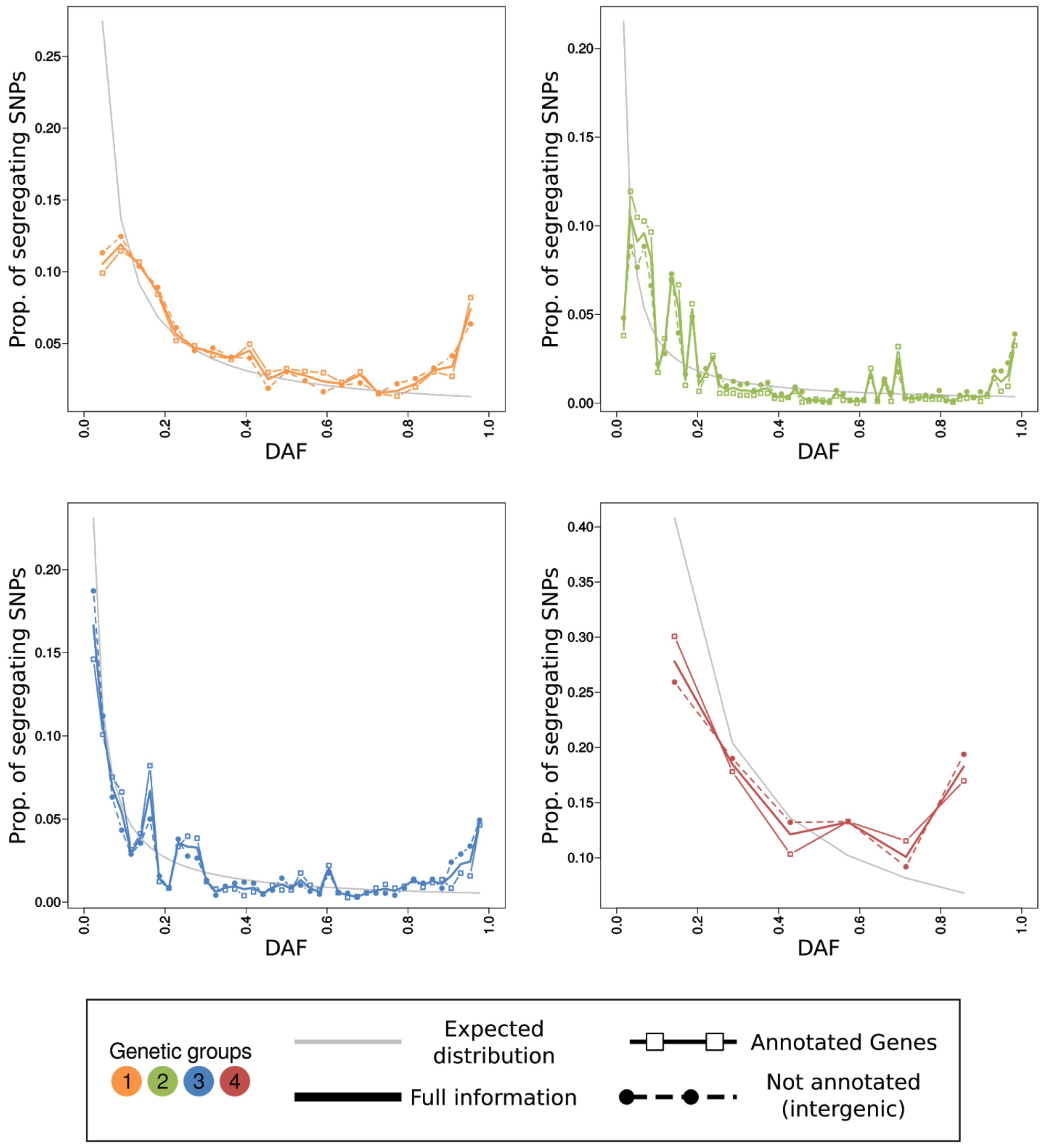

Figure S3. The unfolded Site Frequency Spectrum (SFS). The SFS was calculated independently for each genetic group using genome-wide segregating sites, or only segregating sites within annotated genes or intergenic regions (see inset).The ancestral allele was ascertained using two different outgroups (see Methods section). The gray line shows the expected distribution of the SFS assuming no linked selection.

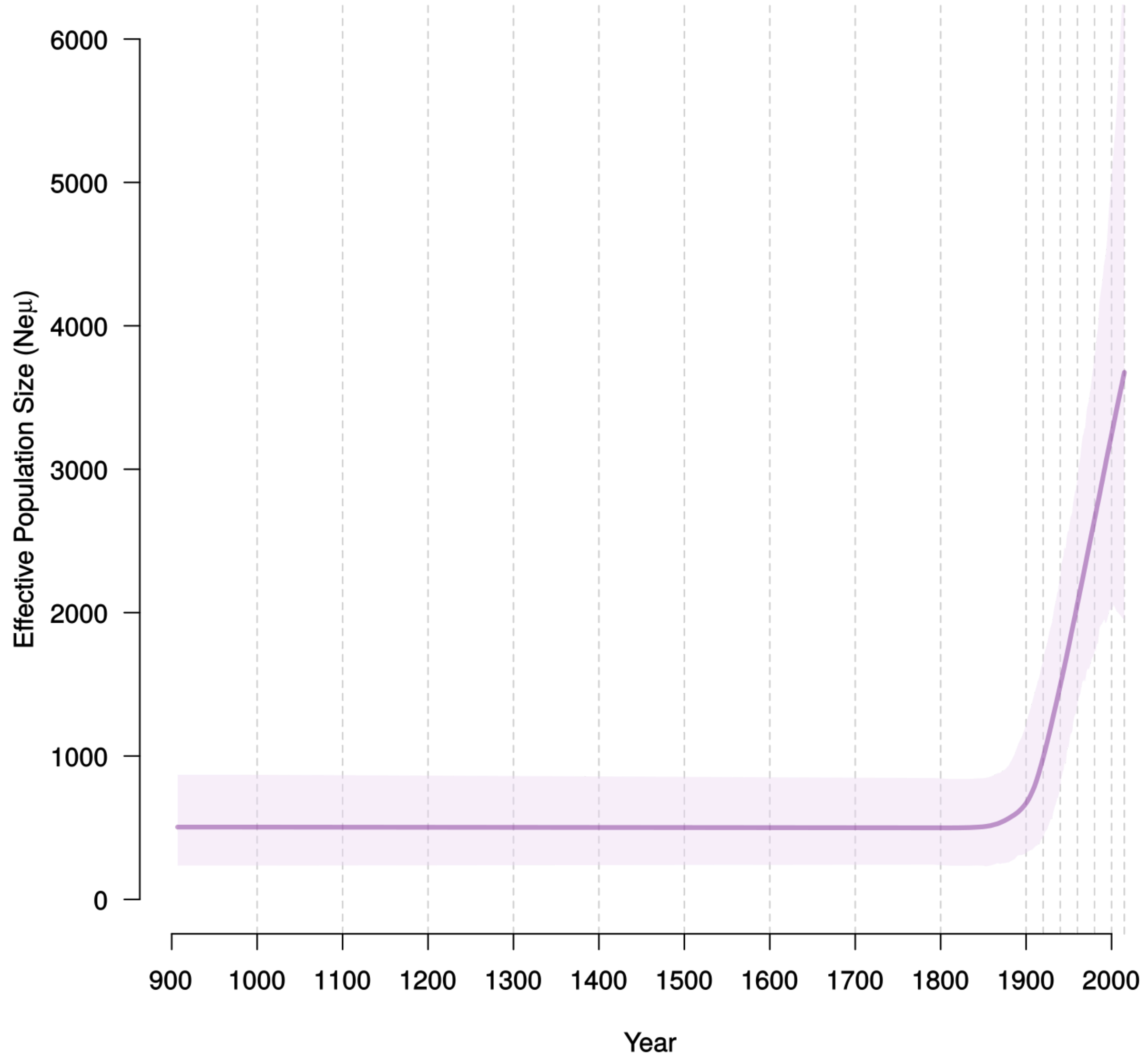

Figure S4. Recent increase of population size in clonal lineages of *Magnaporthe oryzae*. An Extended Bayesian Skyline Plot was calculated from the Bayesian tip calibrated phylogeny (Fig. 4). The thick line depicts the median Effective Population Size and the light colored silhouette represents its 95 HPD interval.

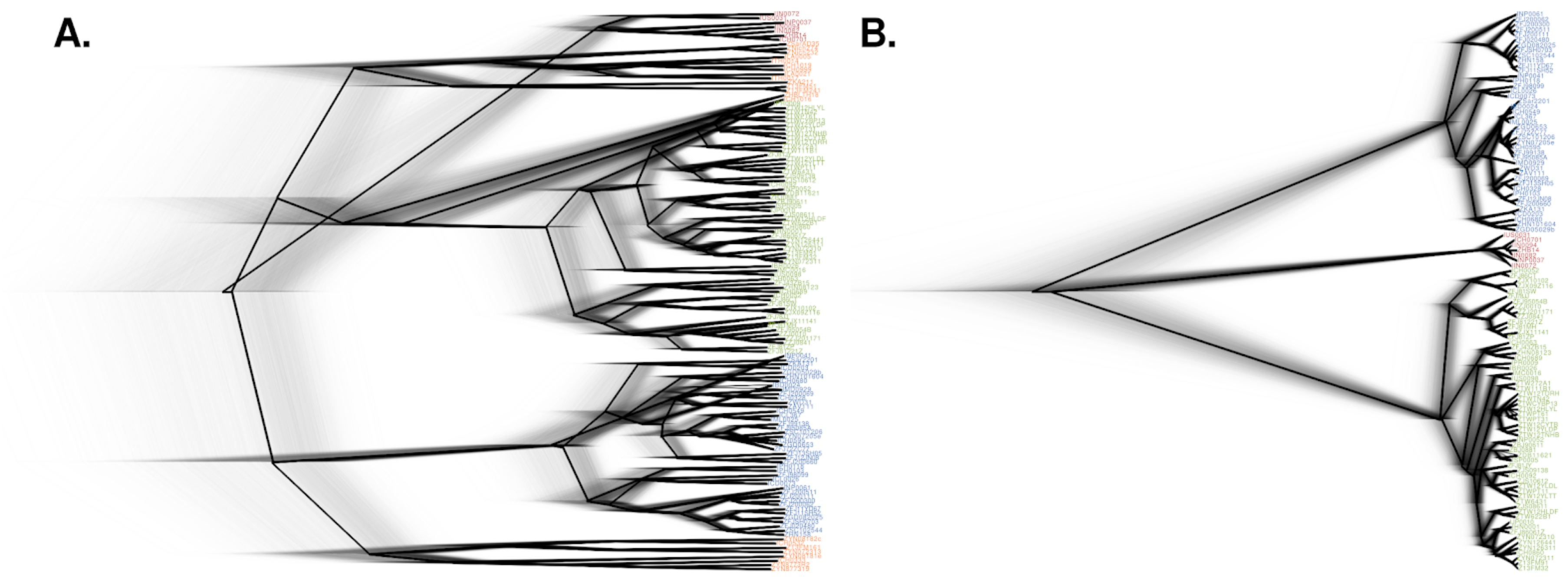

Figure S5. **Effect of recombination on the *Magnaporthe oryzae* phylogeny construction.** Comparison between phylogenies with **(A)** and without **(B)** the diverse recombining group. Grey diffused lines depict all calculated trees, whereas the black lines represent the maximum clade credibility tree.

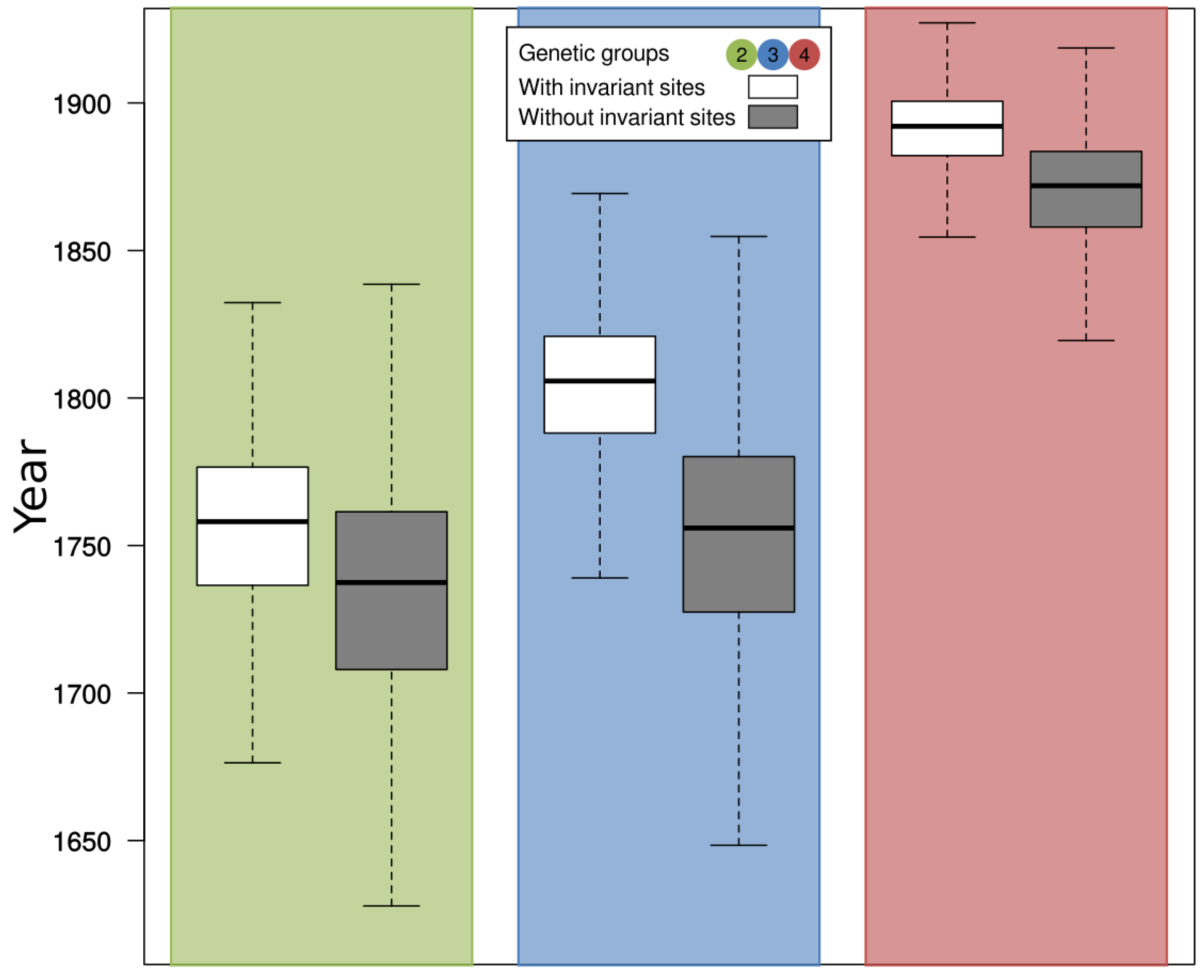

Figure S6. Estimated Time to Most Recent Common Ancestor (TMRCA) for clonal lineages with / without invariant sites.

A.

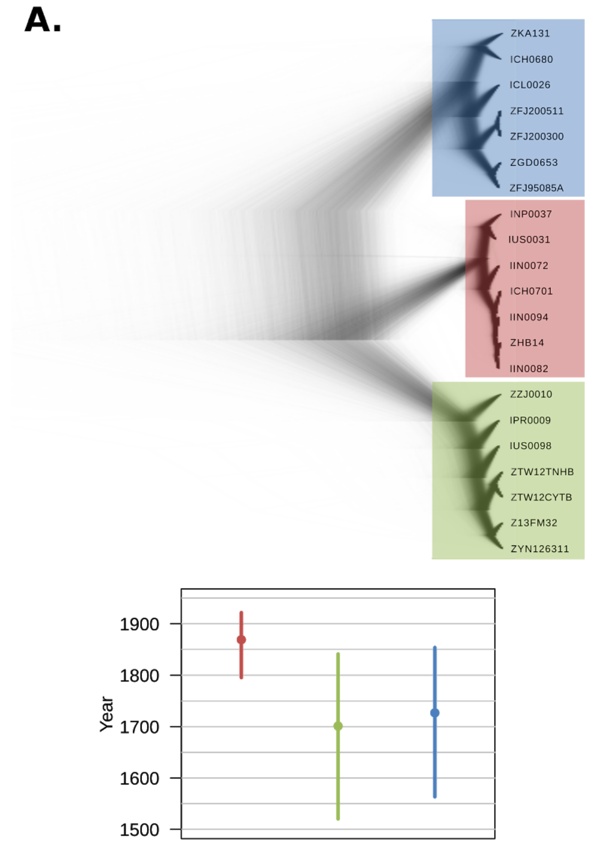

B.

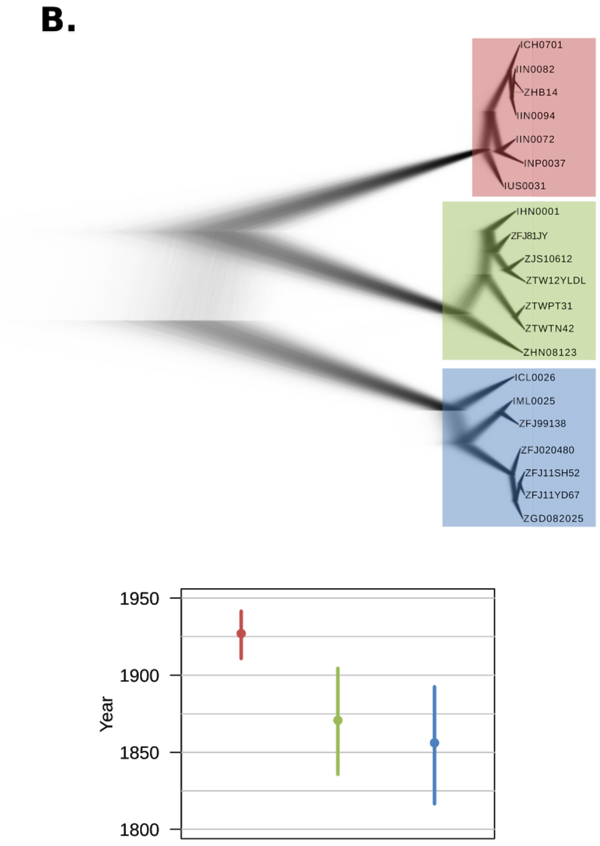

Figure S7. Effect of sample size on the topology of the tree and on the estimation of divergence times. Seven individuals of the genetic groups II and III were randomly sampled in two independent runs (**A**, **B**). Bayesian-based phylogenetic reconstruction was performed using the same described parameters (See Materials and Methods). Bottom panel represents the 95% HPD of the estimated coalescent times and their mean.

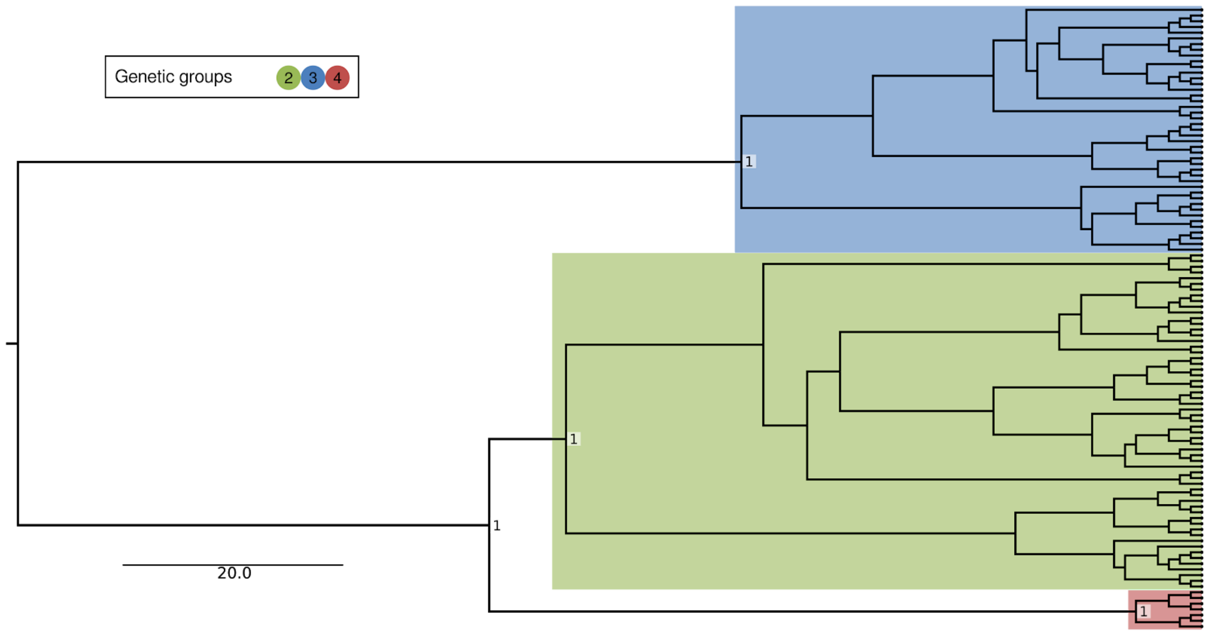

Figure S8. Phylogenetic inference using Single Nucleotide Polymorphisms using SNAPP. Maximum Credibility Tree summarizing the phylogenetic inference after sampling 9,000 trees. The values on the nodes indicate the Bayesian Posterior Probability Support.

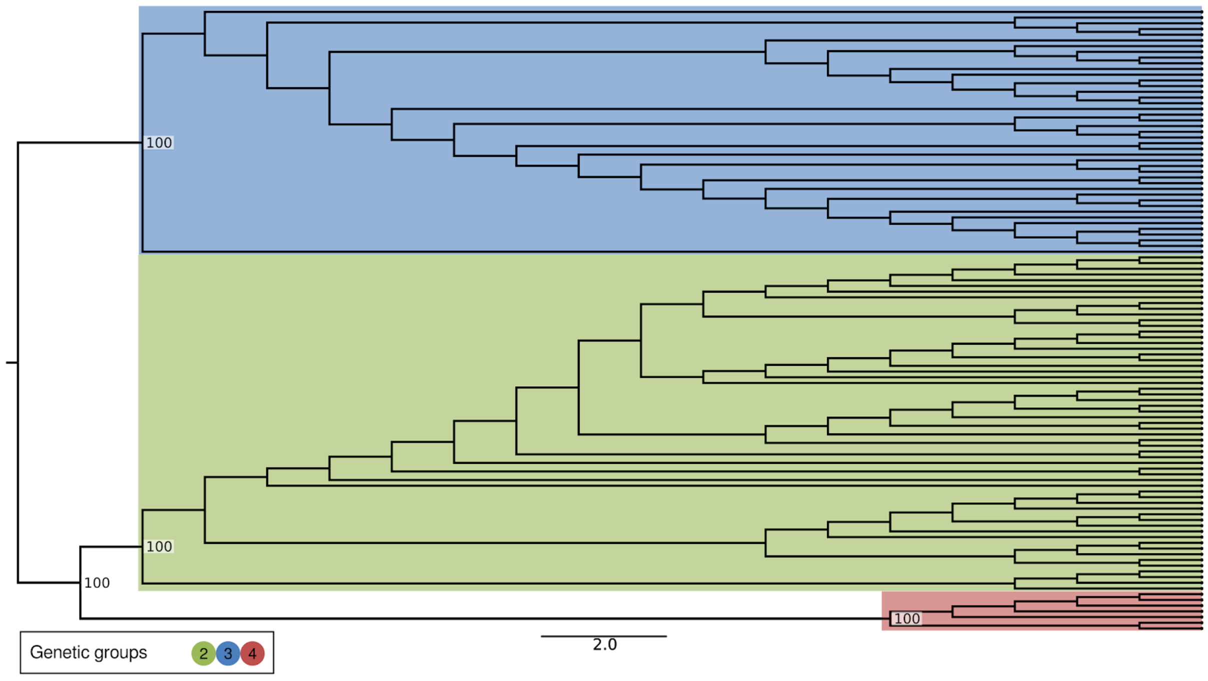

Figure S9. Phylogenetic inference using SVDquartets. The combination of the sets of all possible quartets of isolates is represented in a single phylogenetic tree. The values on the nodes indicate the bootstrap support after 100 replications.

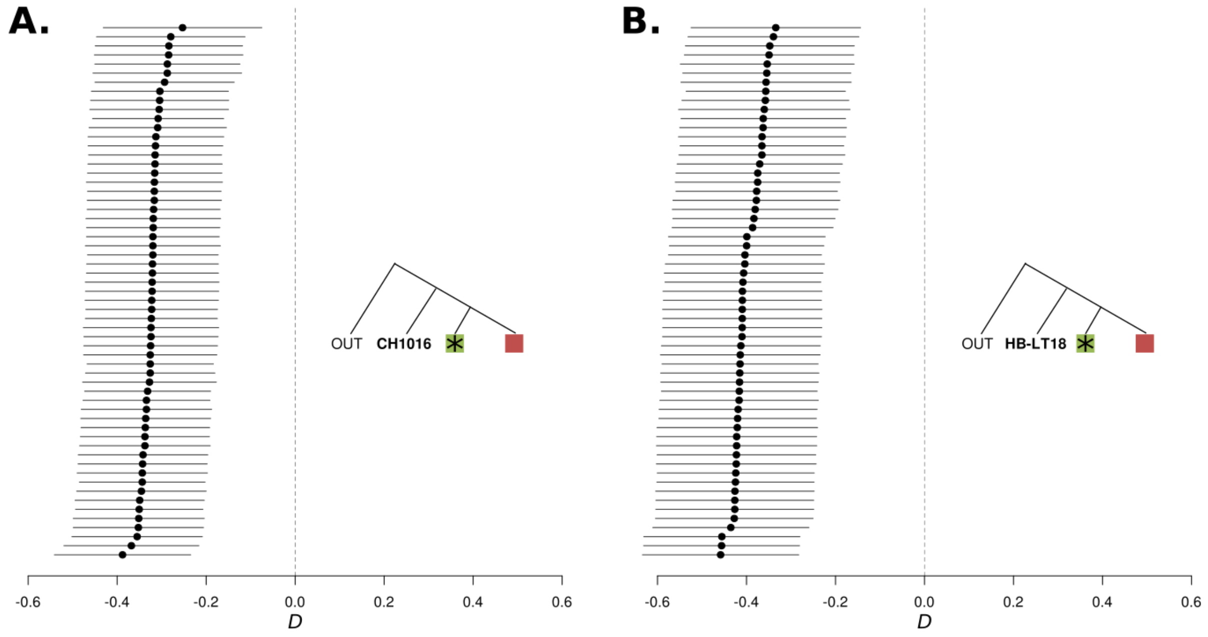

Figure S10. Two Chinese individuals display consistent introgression with the clonal lineage II. *D*-statistics using different phylogenetic configurations depicted as colored inset trees. The green box with the asterisk represents a position in which all individuals from the clonal lineage II were placed in an iterative way. Red boxes represent a fixed individual from the clonal lineage IV. **(A)** *D*(Outgroup, CH1016, Lineage II individual, Lineage IV individual). **(B)** *D*(Outgroup, HB-LT18, Lineage II individual, Lineage IV individual). Points represent *D*-statistic tests, and lines depict 95% confidence intervals.

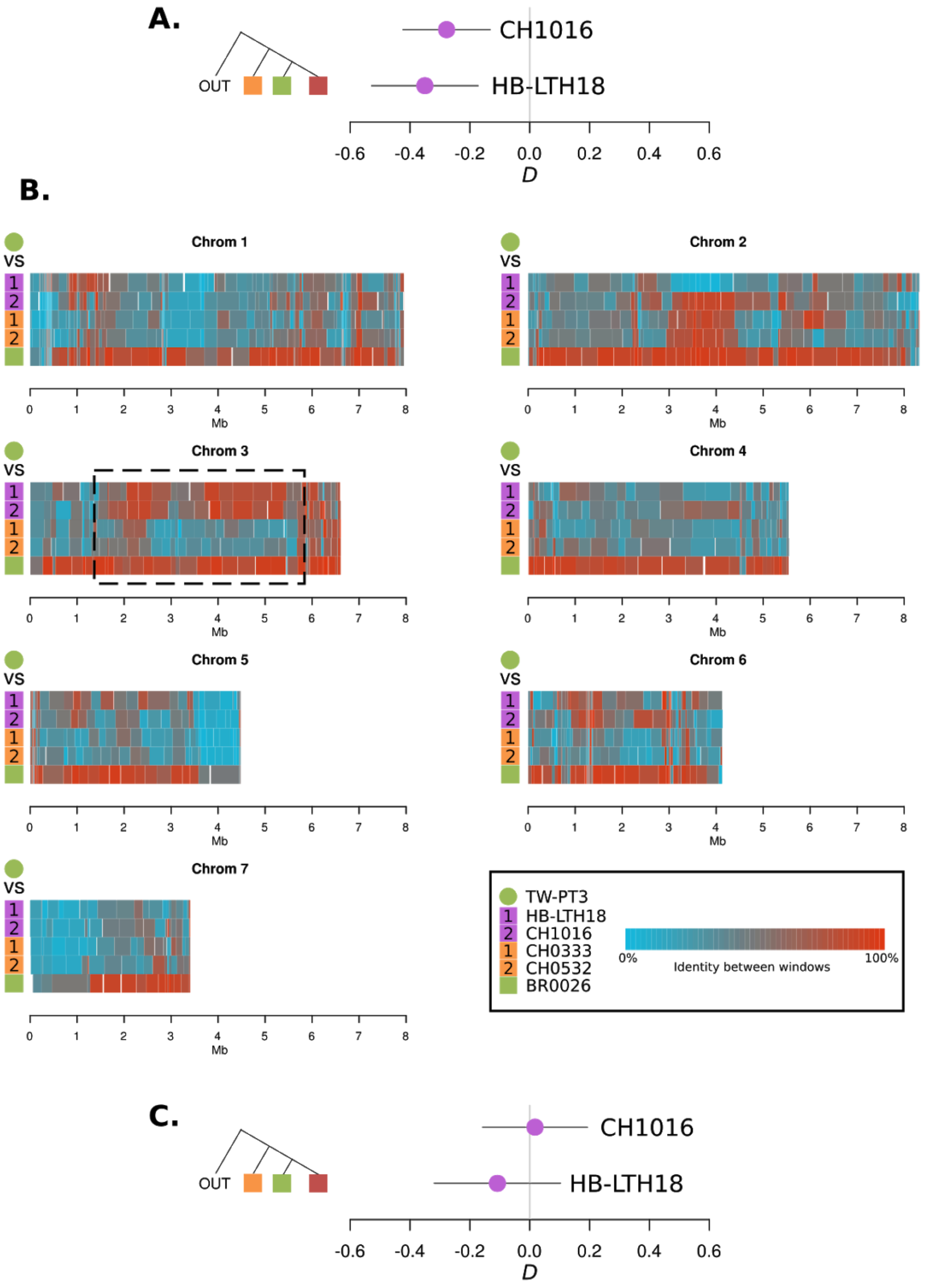

Figure S11. Ancestry-based genomic segmentation of Chinese individuals *CH1016* and *HB-LTH18* reveals a 4 Mb putative introgressed region on chromosome 3. **(A)** The inset tree shows the *D*-statistics configuration, *D*(Outgroup, Orange; Green, Red), used to detect introgression between clonal lineage II and two individuals (*CH1016* and *HB-LTH18*) from the diverse group I (as in Fig 5B). Introgression is inferred based on the significant negative *D*-statistics. **(B)** Each panel shows homologous chromosomes from *CH1016* and *HB-LTH18* in addition to the control individuals from the diverse group I (CH033 and CH0532) and the clonal lineage II (BR0026), segmented according to their ancestry. The color coding represents the level of SNP similarity between each individual and the chosen clonal lineage II individual (TW-PT3) for that particular segment. Chromosome 3 shows a 4 Mb segment inferred to be introgressed between the clonal lineage II and both *CH1016* and *HB-LTH18* (boxed area). **(C)** The same *D*-statistic test as in A. was carried out after all putative introgressed fragments (in red) with a percentage similarity value of >= 60 were removed. The test was not significant, i.e., no different from zero.

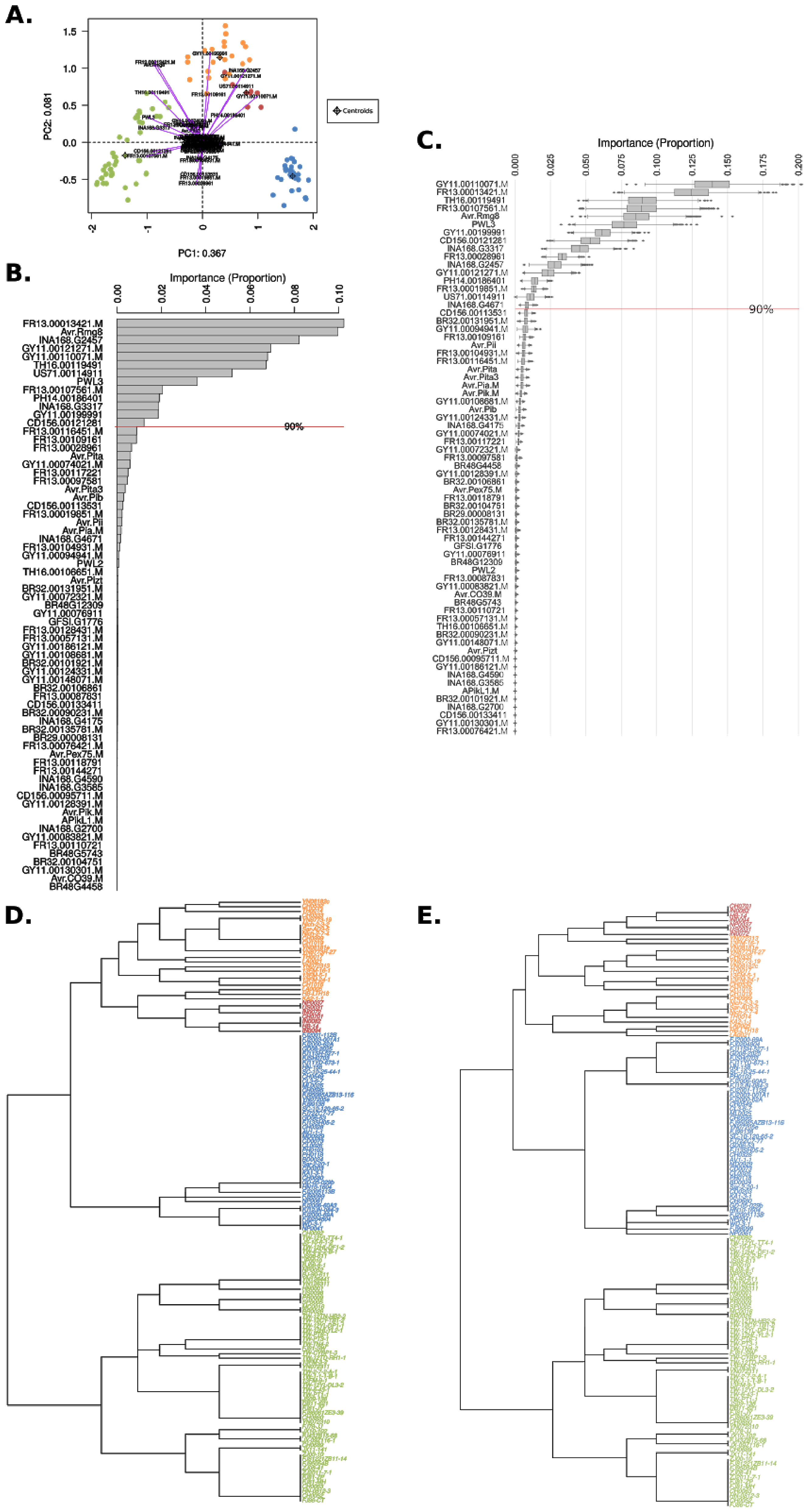

Figure S12. Effector loadings reveal major effector loss in clonal lineage III. **(A)** Biplot based on the presence/ and absence effector matrix (Fig 6C). Dots represent isolates color-coded by their genetic group. Vectors and labels correspond to the effector loadings for PC1 and PC2. All vectors were scaled by 3X for better representation. **(B)** The barplot represents the absolute value of the product of the PC1 and PC2 coordinates for each loading vector. The horizontal red line represents the cumulative sum of 90% of the data. **(C)** The set of boxplots summarize the distribution of the importance of each effector as decision factors for the genetic group assignment under 2,500 iterations of the extremely randomized trees algorithm. The horizontal red line represents the cumulative 90% of the data based on the mean values. **(D)** Hierarchical cluster-based dendrogram built by subsetting the 13 first effectors showed in B. **(E)** Hierarchical cluster-based dendrogram built by subsetting the 16 first effectors showed in **C.**
